# Cognitive reserve over the life course and risk of dementia: a systematic review and meta-analysis

**DOI:** 10.3389/fnagi.2024.1358992

**Published:** 2024-04-12

**Authors:** Yulu Liu, Guangyu Lu, Lin Liu, Yuhang He, Weijuan Gong

**Affiliations:** School of Nursing and School of Public Health, Medical College, Yangzhou University, Yangzhou, China

**Keywords:** cognitive reserve, dementia, life course, meta-analysis, systematic review

## Abstract

**Background:**

The number of people with dementia is soaring. Cognitive reserve has been thought to be associated with dementia risk. It is not clear at which period in the life course and which cognitive reserve proxies contribute to the reduced risk of dementia.

**Methods:**

By scanning four databases (PubMed, Embase, Web of Science, and MEDLINE) up to Jun 3, 2023, longitudinal studies of life-course cognitive reserve and risk of dementia were found. The HRs and 95% CIs for each study were summarized using random effects models. Subgroup analyses and sensitivity analyses were conducted. Utilizing funnel plots, Begg and Egger tests, publication bias was investigated.

**Results:**

A total of 27 studies were included, containing 10 in early-life, 10 in middle-life, and 13 in late-life. All studies used validated questionnaires to measure cognitive reserve, and dementia diagnosis followed recognized worldwide guidelines. All included studies were of medium or low risk. Cognitive reserve in early-life (Hazard ratio (HR): 0.82; 95% confidence interval (CI): 0.79–0.86), middle-life (HR: 0.91; 95% CI: 0.84–0.98) and late-life (HR: 0.81; 95% CI: 0.75–0.88) all have protective effects on dementia risk. Multiple sensitivity analyses showed consistent results.

**Conclusion:**

Dementia risk is reduced by the buildup of cognitive reserves during life-course. Accumulation of proxies for cognitive reserve in early and late life had the greatest effect on dementia risk reduction. Social connection may be an effective approach to lower dementia risk.

## 1 Introduction

The public health challenge of dementia has profound physical, psychological, social, and economic implications for individuals afflicted with the disease and their caregivers, families, and broader society (Aranda et al., 2021; Alzheimer’s Association, 2022). This disease stands as the leading cause of disability among older adults globally. As of 2015, approximately 50 million people globally were affected by dementia, constituting about 5% of the elderly population. Projects indicate that by 2030, the number of individuals with dementia will rise to 82 million, and by 2050, to 152 million (WHO, 2019). Given the current absence of effective treatment for dementia, it is crucial to identify pertinent risk factors and implement appropriate preventative measures (WHO, 2019).

According to research on dementia prevention (WHO, 2019; Livingston et al., 2020), cognitive reserve (CR) offers comprehensive protection against the onset of this disease. The concept of CR was initially proposed by Stern (2002), referring to the adaptability of cognitive processes. This adaptability explains why certain cognitive capacities or daily functioning are more or less susceptible to brain aging, pathology, or injury (Stern et al., 2020). This phenomenon may elucidate why some patients display dementia-related brain damage without accompanying clinical symptoms (Livingston et al., 2020). CR is a dynamic construct influenced by both intrinsic factors, such as intelligence quotient (IQ), and lifelong exposure (Stern et al., 2020). Directly assessment of CR is challenging because it is shaped by the accumulation of several protective and risk factors. The use of proxies for CR is still controversial. Researchers usually use proxy indicators such as education, occupation, cognitive activity, and social engagement to assess CR (Stern, 2009). However, as the protective role of lifestyle factors against dementia risk is gradually being explored, factors such as physical activity (Reas et al., 2019) have also been used to assess the relationship with cognitive function and are discussed together with CR-related indicators (Najar et al., 2019). Although physical activity may also be one of the favorable factors to reduce the risk of dementia according to existing studies (Palta et al., 2019; Hwang et al., 2023), we preferred to use the original concept of CR proposed by Stern (2009) in this study considering the rigor of the study. Referring to the Reserve and Resilience meetings sponsored by the NIA, we identified the CR proxies in this study as occupational complexity, educational attainment, leisure activities, cognitive activities, and social connections, and other measures (e.g., socioeconomic status) that could reflect the above proxies were also taken into account if they appeared.

Recent studies have identified certain CR traits associated with a lower risk of dementia and slower rates of memory decline during natural aging (Wang et al., 2019; Ding et al., 2020; Foverskov et al., 2020; Duffner et al., 2022). However, other studies have reported no statistically significant difference in dementia risk associated with these CR proxies (Prince et al., 2012; Sörman et al., 2014; Dekhtyar et al., 2016; Rusmaully et al., 2017; Takasugi et al., 2019; Zhang et al., 2021). Additionally, preventing dementia is a long-term process, and the impact of the effect of CR on dementia risk may vary across different life stages (Xu et al., 2019). While a meta-analysis (Hyun et al., 2022) established a link between education and occupational complexity in early and middle life and the risk of dementia, there still exists a dearth of literature on the relationship between various CR proxies and dementia risk at each stage of the lifespan. The point in the life course and the specific CR proxies that contribute to dementia risk reduction remains unclear. This review plans to investigate the relationship between CR levels and the risk of dementia at various life stages and provide a foundation for early dementia detection and prevention.

## 2 Methods

This systematic review and meta-analysis was registered in the International Prospective Register of Systematic Reviews (PROSPERO) with the registration number CRD42022330713. The review procedure was governed by the Preferred Reporting Items for Systematic Reviews and Meta-Analyses (PRISMA) checklist (Page et al., 2021).

### 2.1 Search strategy

A computerized systematic literature search was performed in PubMed, Embase, Web of Science, and MEDLINE databases, from their inception to June 3, 2023. The search terms included a combination of keywords related to CR, dementia, and risk. For CR, the following keywords were used: cognitive reserve OR brain reserve OR cognitive capacity OR neural reserve OR brain maintenance OR cognitive resilience OR brain resilience OR education OR occupation OR leisure activity OR cognitive activity OR social connection. The dementia-related keywords included dementia OR dement OR Alzheimer. Lastly, the following keywords were applied for risk: risk OR hazard ratio (HR). The search was limited to human studies and full-text original articles, with no language restrictions. The full search strategy is provided in Supplementary Appendix A.

### 2.2 Eligibility criteria

Studies were required to meet the following inclusion criteria: (a) Evaluation of CR in early, middle, and later life using CR proxy indicators (Stern, 2009), which encompassed measures of socioeconomic status (e.g., occupational complexity, educational attainment, leisure activity, cognitive activity, and social connection). For this study, early life was defined as birth to 30 years old, middle life as 30–60 years old, and late-life as over 60 years old; (b) Analysis of the relationship between life course CR accumulation and the risk of dementia; and (c) Utilization of a longitudinal study design. Studies were excluded if: (a) Participants exhibited dementia or other neurological diseases at baseline; (b) Participants had mental disorders at baseline; (c) Non-human subjects were included; and (d) Papers contained incomplete information, systematic reviews, meta-analyses, or reports from meetings or congresses.

### 2.3 Study selection

The searched articles were input into Rayyan (Ouzzani et al., 2016), a web-based tool designed for the efficient management of selected articles. Following the predefined inclusion and exclusion criteria, two reviewers independently conducted an initial screening of the titles and abstracts. Articles that passed this initial screening underwent a secondary screening upon acquisition of the full text to determine their eligibility for inclusion.

### 2.4 Quality assessment

The Newcastle-Ottawa scale (NOS) (Stang, 2010), a well-established method for assessing the quality of cohort studies, was utilized to evaluate the quality of these studies from three perspectives: selection, comparability, and outcome. Each part has a corresponding score, and the standards set by the Agency for Healthcare Research and Quality (AHRQ) (Wells et al., 2021) were applied to the NOS score. According to the scores for each section, the articles’ quality ranged from good to fair to poor.

### 2.5 Data extraction

Data extraction was performed independently by two investigators, with any discrepancies resolved through discussion with a third researcher to reach a consensus. Information extracted from the selected studies included publication details (author, year, and country), participant characteristics (age, gender, and sample size), cohort details (source and follow-up period), exposure information (measurement of CR), outcome data (dementia diagnosis), and adjusted factors. Summary statistics, such as HR and their corresponding 95% confidence intervals (CIs), were recorded. In instances of missing data, attempts were made to contact the original authors for retrieval.

### 2.6 Data synthesis

Considering that some commands exceeded the capacity of Stata SE 15 while others did not meet the requirements of Stata MP 17, the statistical analysis of the findings from the included studies was conducted using both Stata SE 15 and Stata MP 17 (Stata Corp LP, USA). A narrative synthesis of the findings from the included studies was performed. Additionally, a meta-analysis was conducted using the inverse variance weighted method, pooling the extracted Hazard ratios (HR) and their corresponding 95% confidence intervals using a random effects mode (Borenstein et al., 2010). Statistical heterogeneity was quantified using the I^2^ statistic (Jonathan et al., 2022). Publication bias was assessed using funnel plots and Begg and Egger tests (Egger et al., 1997).

### 2.7 Sensitivity analyses

To assess the stability and reliability of the results, sensitivity analyses were conducted as follows: (a) The stability of the overall results was verified by analyzing the same data using both fixed-effect and random-effect models; (b) The impact of individual studies on the overall results was assessed by calculating pooled risk estimates and heterogeneity after sequentially removing each study from the analyses; (c) To address the potential influence of CR Proxies on the results, the studies were stratified based on different categories in early-life, middle-life, and late-life; (d) To evaluate the influence of publication bias and subjective judgment in the inclusion and exclusion of literature on the results, the trim-and-fill method was employed; and (e) To consider the potential effect of study quality on the results, the analysis was restricted to studies with a low risk of bias.

## 3 Results

### 3.1 Search results

**Figure 1:**
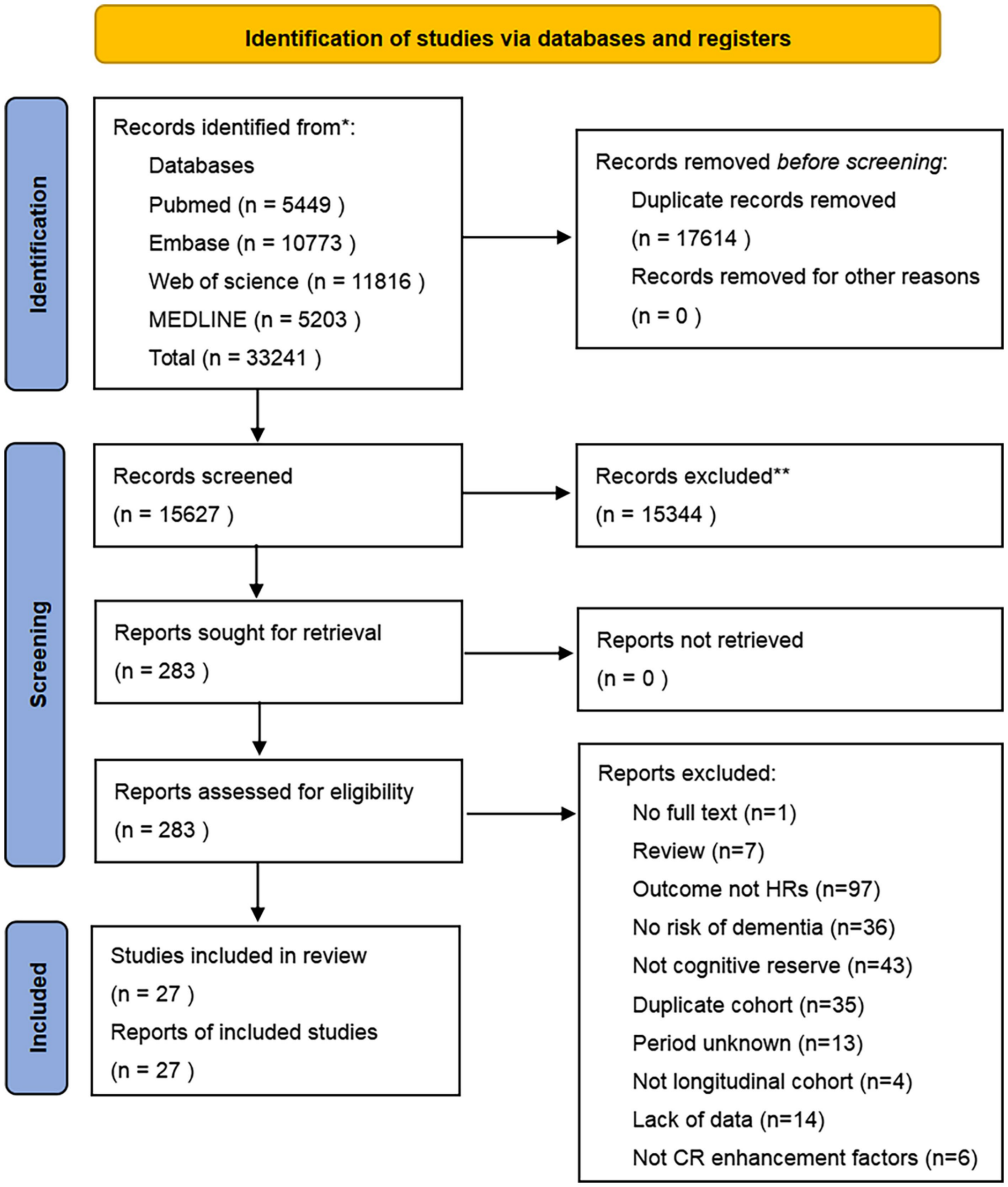
PRISMA flow diagram.

Figure 1 illustrates the various phases of the systematic search and article selection during the literature review process. A total of 33,241 papers were identified for this review. Following deduplication, 15,627 potentially relevant citations were identified for screening of titles and abstracts. Subsequently, 283 full-text papers were selected for in-depth analysis. Based on the inclusion and exclusion criteria, 256 papers were excluded. The reasons for exclusion are detailed in the PRISMA flowchart (Figure 1) and Supplementary Appendix Table A1. Ultimately, 27 papers met the criteria for inclusion in the systematic review and meta-analysis. All included papers were longitudinal studies that examined the risk of dementia as their primary outcome and reported their results as HRs.

### 3.2 Characteristics of included studies

Detailed characteristics of the included studies are presented in Tables 1–3. In total, 27 articles were included, with 10 focusing on early-life CR, 10 on middle-life CR, and 13 on late-life CR. The publication years of the included studies ranged from 2004 to 2023, with 70.4% published in the last decade and 44.4% in the last 5 years. The sample sizes of the individual studies varied widely, ranging from 176 to 1,341,842 participants. The proportion of females in studies including both sexes ranged from 29.7 to 76.0%, while four articles exclusively included males. The studies adjusted for multiple potential confounders, including age, sex, education, race/ethnicity, moderate drinking, current smoking, healthy diet score, obesity, diabetes, hypertension, depression, social isolation, hearing problems, regular physical activity, high cholesterol, cardiovascular disease, and traumatic brain injury, among others.

**Table 1:** Detailed characteristics of studies on early-life CR and risk of dementia.

No.	Study	Year	Country	Cohort	Age	Female%	Follow-up period	Total participants	Measurement of cognitive reserve	Dementia diagnosis	Adjusted
1	Ma^#^	2022	UK	The UK Biobank Study	≥60	51.9	8.9y	174,161	Education: certificate and degree	Medical history	Age, sex, Townsend deprivation index, moderating drinking, current smoking, healthy diet score, obesity, diabetes, hypertension, depression, social isolation, hearing problem, regular physical activity, high cholesterol, cardiovascular disease, and traumatic brain injury
2	Dominika	2021	Sweden	18 full birth cohorts (1920–1937)	≥65	51.4	31y	1,341,842	Education: certificate and degree	ICD-9, ICD-10	Sex
3	Foverskov	2020	Danmark	DCD	≥60	0	7.7y	477,442	Education: certificate and degree	ICD-8, ICD-10	Birth year, age, cognitive ability, education
4	Lamballais	2020	Netherlands	The Rotterdam Study	66.3 ± 8.7	55.4	6y	4,112	Education: certificate and degree	DSM-III-R	Cohort, sex, age difference between cognitive testing and MRI scan, hypertension, alcohol intake, smoking status, BMI, and APOE ε4 allele count
5	Takasugi^#^	2019	Japan	JAGES	≥65	53.6	6y	52,063	Education: certificate and degree	Medical history	Age, marital status, living arrangement, occupational status, depression symptoms, stroke, hypertension, diabetes, alcohol consumption, smoking, daily walking time, educational attainment, the longest job held, and equivalised income
6	Rusmaully^#^	2017	UK	Whitehall II	75.6 ± 4.6	29.7	16.1y	7,499	Education: certificate and degree	ICD-10	Age, sex, ethnicity, time-dependant marital status, 5-year birth cohort, smoking, alcohol consumption, physical activity, and fruit and vegetable consumption, hypertension, diabetes, use of medication for cardiovascular disease, anxiety and depression symptoms, cardiovascular disease, and chronic obstructive pulmonary disease.
7	Dekhtyar	2016	Sweden	The Kungsholmen Project (KP)	≥75	75.7	5y	440	Education: certificate and degree	DSM-III-R	Age, gender, childhood school grades, education, occupational complexity
8	Then	2016	Germany	LEILA75+	≥75	70.4	15y	983	Education: certificate and degree	ICD-10, DSM-III-R	Age, gender, marital status, living situation, diabetes, heart attack, stroke, history of depression
9	Dekhtyar	2015	Sweden	The Uppsala Birth Cohort Multigenerational Study	≥65	46.3	21y	7,574	Education: certificate and degree	Medical history	Sex, birth cohort, year of the follow-up, childhood school grades, formal educational attainment, and occupational complexity
10	Yaffe^#^	2013	USA	The Health, Aging, and Body Composition study	73.6	50.2	12y	2,457	Education: certificate and degree	Medical history	Demographics, apolipoprotein E e4 status, lifestyle, and socioeconomic status (education, literacy, income, perceived financial adequacy)

^#^Use the multiplicative inverse of the reported HR.

DSM, The Diagnostic and Statistical Manual of Mental Disorders; NINDS, National Institute of Neurological Disorders and Stroke; ICD, International Classification of Diseases.

**Table 2:** Detailed characteristics of studies on middle-life CR and risk of dementia.

No.	Study	Year	Country	Cohort	Age	Female%	Follow-up period	Total participants	Measurement of cognitive reserve	Dementia diagnosis	Adjusted
1	Hwang^#^	2023	USA	FHS (Gen 2)	45–65	51.7	21y	2,476	Physical activity: the Physical Activity Index Social network contact: number of social ties	DSM-IV	Age, sex, APOE ε4, and history of cardiovascular events
2	Yang	2023	China	A cohort of an AD high-risk population	≥60	52.2	2y	1,545	Occupation: the primary lifetime occupation prior to retirement	Guidelines group of Alzheimer’s Disease Branch of ADC, 2021	Age, gender, and educational degree
3	Hyun	2020	USA	EAS	≥70	61.6	4.6y	1,079	Occupation: the Dictionary of Occupational Titles	DSM-IV	Retirement age, sex, race/ethnicity, education, income, vascular and other comorbidities
4	Sommerlad	2019	UK	Whitehall II	35–55	33.1	28.6y	10,228	Social network contact: the Berkman-Syme social network index	ICD-10	Age, sex, education, social class, ethnicity, smoking, alcohol, exercise, employment status and marital status
5	Takasugi^#^	2019	Japan	JAGES	≥65	53.6	6y	52,063	Occupation: the primary lifetime occupation prior to retirement	Medical history	Age, marital status, living arrangement, occupational status, depression symptoms, stroke, hypertension, diabetes, alcohol consumption, smoking, daily walking time, educational attainment, the longest job held, and equivalised income
6	Rusmaully^#^	2017	UK	Whitehall II	75.6 ± 4.6	29.7	16.1y	7,499	Occupation: the British Civil Service grade of employment	ICD-10	Age, sex, ethnicity, time-dependant marital status, 5-year birth cohort, smoking, alcohol consumption, physical activity, and fruit and vegetable consumption, hypertension, diabetes, use of medication for cardiovascular disease, anxiety and depression symptoms, cardiovascular disease, and chronic obstructive pulmonary disease.
7	Dekhtyar	2016	Sweden	The Kungsholmen Project	≥75	75.7	5y	440	Occupation: the primary lifetime occupation prior to retirement	DSM-III-R	Age, gender, childhood school grades, education, occupational complexity
8	Dekhtyar	2015	Sweden	The Uppsala Birth Cohort Multigenerational Study	≥65	46.3	21y	7,574	Occupation: Swedish occupational codes	Medical history	Sex, birth cohort, year of the follow-up, childhood school grades, formal educational attainment, and occupational complexity
9	Kroeger	2008	Canada	CSHA	≥65	51.2	9.3y	3,557	Occupation: the 1980 Canadian Standard Occupational Classification	DSM-III-R	Sex, education, work-related physical activity, leisure physical exercise, hobby, alcohol consumption, smoking, family history of dementia, history of hypertension, history of diabetes mellitus, and history of coronary heart disease
10	Saczynski	2006	USA	The Honolulu Heart Program	53.8 (median)	0	4.1y	2,513	Social network contact: marital status, living arrangement, participation in social, political, or community groups, participation in social events with coworkers, and the existence of a confidant relationship	DSM-III-R	Age, education, Cognitive Abilities Screening Instrument score, APOE ε4, cerebrovascular disease, coronary heart disease, depression, and disability

^#^Use the multiplicative inverse of the reported HR.

DSM, The Diagnostic and Statistical Manual of Mental Disorders; ICD, International Classification of Diseases; CDR, the Clinical Dementia Rating Scale; NINDS, National Institute of Neurological Disorders and Stroke.

**Table 3:** Detailed characteristics of studies on late-life CR and risk of dementia.

No.	Study	Year	Country	Cohort	Age	Female%	Follow-up period	Total participants	Measurement of cognitive reserve	Dementia diagnosis	Adjusted
1	Hwang^#^	2023	USA	FHS (Gen 2)	66–80	47.8	21y	460	Physical activity: the Physical Activity Index Social connection: number of social ties	DSM-IV	Age, sex, APOE ε4, and history of cardiovascular events
2	Kallianpur^#^	2022	USA	Kuakini HAAS	71–93	0	10y	2,636	Social connection: the 10-item LSNS	DSM-III-R	Age, education, APOE ɛ4, prevalent stroke, depressive symptoms, and CASI score
3	Tian	2022	China	CLHLS	≥65	57.3	10y	11,821	Cognitive activity: by asking “Do you now perform the following activities (playing cards/mahjong) regularly?”	Medical history	Age, sex, education, household income, marital status, smoking status, drinking status, exercise, BMI, living with family members, hypertension, diabetes, and MMSE score
4	Duffner	2022	England	ELSA	≥60	54.9	9.8y	7,917	Cognitive activity: collected by questionnaires Social connection: the membership of various clubs or societies was assessed	Medical history	Age, sex, education, wealth, LIBRA_adj_
5	Nemoto	2017	Japan	AGES	≥65	48.9	7.9y	9,234	Social connection: the Japanese General Social Survey	Medical history	Sex, age, educational attainment, marital status, living arrangement, occupational status, walking time, medical history, alcohol consumption, smoking, depression, and IADL
6	Grande	2014	Italy	RTCDUM Research	74 (median)	58.0	2.59y	176	Cognitive activity: lifestyle questionnaire	DSM-IV	Age, gender, education, MMSE score, Geriatric Depression Scale score, MCI subtype, APOE genotype, Physical activity score, Cognitive activity score, Social activity score
7	Sörman	2014	Sweden	The Betula prospective cohort study	≥65	56.7	15y	1,475	Cognitive activity: leisure activity questionnaire	DSM-IV	Age, gender, education, diseases, smoking, alcohol use, marital status, general stress, feelings of depression, and APOE genotype
8	Dartigues	2013	France	The Paquid cohort	≥65	58.0	20y	3,670	Cognitive activity: a standardized questionnaire during a face-to-face interview	DSM-III-R	Age, gender, education, marital status, history of stroke, diabetes, MMSE score and depression
9	Buchman	2012	USA	The Memory and Aging Project	81.6 (mean)	76.0	4y	716	Cognitive activity: 6 items about activities involving social interaction over the past year Social connection: 7 cognitive activities over the past year	NINDS	Age, sex, and education, level of total daily physical activity, self-report physical activity, the frequency of social and cognitive activities
10	Hughes	2010	USA	MoVIES	≥65	66.5	6.1y	942	Cognitive activity: reading books, magazines, and newspapers, and engaging in hobbies including board games, crafts, crossword puzzles, jigsaw puzzles, musical instruments, bridge, and other card games	CDR	Age, gender, education, depressive symptoms, physical exercise, functional impairment, self-reported health, medication use, and recruitment status
11	Akbaraly	2009	France	The Three-City cohort study	≥65	60.9	4y	5,698	Cognitive activity: 2 different self-report frequency questionnaires, 1 for daily and 1 for monthly	DSM-IV	Gender, educational level, occupational grade, study center,marital status, hypertension, diabetes, vascular diseases history, hypercholesterolemia, depressive symptoms, APOE genotype, incapacity in daily life activity, and cognitive impairment assessed by the MMSE
12	Saczynski	2006	USA	The Honolulu Heart Program	76.8 (median)	0	4y	2,513	Social network: marital status, living arrangement, participation in social, political, or community groups, number of face-to-face or telephone contacts with close friends per month, and the existence of a confidant relationship	DSM III-R	Age, education, Cognitive Abilities Screening Instrument score, APOE ɛ4, cerebrovascular disease, coronary heart disease, depression, and disability
13	Bal	2004	–	–	75–85	64.0	5.1y	469	Cognitive activity: six mental activities (reading, writing for pleasure, crossword puzzles, board or card games, organized group discussions, and playing musical instruments) and frequency of participation in each	DSM-III-R	Age, sex, education level, chronic medical illness, and baseline cognitive status

DSM, The Diagnostic and Statistical Manual of Mental Disorders; ICD, International Classification of Diseases; CDR, the Clinical Dementia Rating Scale; NINDS, National Institute of Neurological Disorders and Stroke.# Use the multiplicative inverse of the reported HR.

### 3.3 Measurement of life course cognitive reserve

In the included studies, early-life CR proxies primarily consisted of education. Proxies for midlife CR included occupation complexity and social network contact, while proxies for CR in late-life encompassed cognitive activity and social connection. Given the diverse geographical origins of the study populations, variations in educational attainment classifications may exist, potentially leading to partial heterogeneity when combining results. To address this, the study classified the lowest level of education into two categories: primary and junior high school education. Participants with less than 7 years of education were categorized as having primary education, while those with less than 10 years were categorized as having junior high school education. This standardization facilitated subsequent meta-analysis. CR proxies were assessed using validated questionnaires in all studies, with the lowest level of each CR proxy indicator used as a reference category for comparison with the highest level. When the highest level was used as a reference category, the inverse of the HR and its corresponding 95% CI were utilized in the data analysis. Detailed measurements of life course CR are provided in Tables 1–3.

### 3.4 Diagnosis of dementia

All the included studies diagnosed dementia according to accepted standards, such as The Diagnostic and Statistical Manual of Mental Disorders (DSM-III, DSM-III-R, DSM-IV, DSM-5) (Wakefield, 2016), International Classification of Diseases (ICD) (The Lancet, 2019), Clinical Dementia Rating (CDR) (Morris, 1993), National Institute of Neurological Disorders and Stroke (NINDS), and Guidelines group of Alzheimer’s Disease Chinese (ADC) 2021.

### 3.5 Quality assessment and potential bias

The quality assessment results are presented in Table 4. The study quality scores ranged from 6 out of 9 to 9 out of 9. Among the 27 studies, 21 were assessed as having a low risk of bias, while 6 studies were categorized as having a moderate risk of bias. Notably, some gender-limited studies incurred a reduction in quality within the selection domain. Additionally, all studies utilized self-assessment of exposure, which contributed to reduced quality in the selection domain, except for one study (Dartigues et al., 2013) that employed structured interviews. Furthermore, given the longitudinal nature of the included studies, there was a certain risk of loss to follow-up, resulting in a lower score in the outcome domain. Specifically, 9 out of 10 studies analyzing early-life CR and the risk of dementia exhibited a low bias, while 8 out of 10 studies analyzing midlife CR and the risk of dementia demonstrated a low bias. Lastly, 10 out of 13 studies analyzing late-life CR and the risk of dementia had a low bias.

**Table 4:** Assessment of quality and risk of bias according to the Newcastle-Ottawa scale.

No.	Author	Selection/4	Comparability/2	Outcome/3	Total/9	Quality assessment	Risk of bias
1	Ma 2022	4	2	2	8	Good	Low
2	Duffner 2022	3	2	3	8	Good	Low
3	Nemoto 2017	3	2	2	7	Good	Low
4	Hyun 2020	3	2	2	7	Good	Low
5	Sommerlad 2019	2	2	2	6	Fair	Moderate
6	Dekhtyar 2016	3	1	2	6	Good	Low
7	Kroeger 2008	3	2	2	7	Good	Low
8	Rusmaully 2017	2	2	2	6	Fair	Moderate
9	Foverskov 2020	2	2	2	6	Fair	Moderate
10	Then 2016	3	2	2	7	Good	Low
11	Saczynski 2006	2	2	3	7	Fair	Moderate
12	Hughes 2010	3	2	2	7	Good	Low
13	Grande 2014	3	2	3	8	Good	Low
14	Akbaraly 2009	3	2	3	8	Good	Low
15	Bal 2004	2	2	2	6	Fair	Moderate
16	Sörman 2014	3	2	2	7	Good	Low
17	Dekhtyar 2015	3	2	2	7	Good	Low
18	Dartigues 2013	4	2	3	9	Good	Low
19	Lamballais 2020	3	2	3	8	Good	Low
20	Takasugi 2019	4	2	2	8	Good	Low
21	Buchman 2012	3	2	3	8	Good	Low
22	Hwang 2023	3	2	3	8	Good	Low
23	Tian 2022	3	2	2	7	Good	Low
24	Kallianpur 2022	2	2	3	7	Fair	Moderate
25	Yang 2023	3	2	3	8	Good	Low
26	Dominika 2021	4	2	2	8	Good	Low
27	Yaffe 2013	3	2	2	7	Good	Low

The funnel plots depicting the association of early-life, middle-life, and late-life CR with the risk of dementia are presented in Supplementary Appendix B1. Results from the Begg and Egger tests indicated no evidence of publication bias for the early-life period (*p* = 0.858 and 0.757). However, a statistically significant difference was observed for the middle-life period (*p* = 0.049 and 0.099) and late-life period (*p* = 0.729 and 0.001), suggesting potential publication bias. Additionally, the trim-and-fill method was employed as a supplementary analysis, but no studies were filled, indicating the stability of the findings (Supplementary Appendix B5).

### 3.6 Study findings

All of the studies evaluated the association between longitudinal changes in CR and the risk of dementia.

#### 3.6.1 Early-life CR and risk of dementia

**Figure 2:**
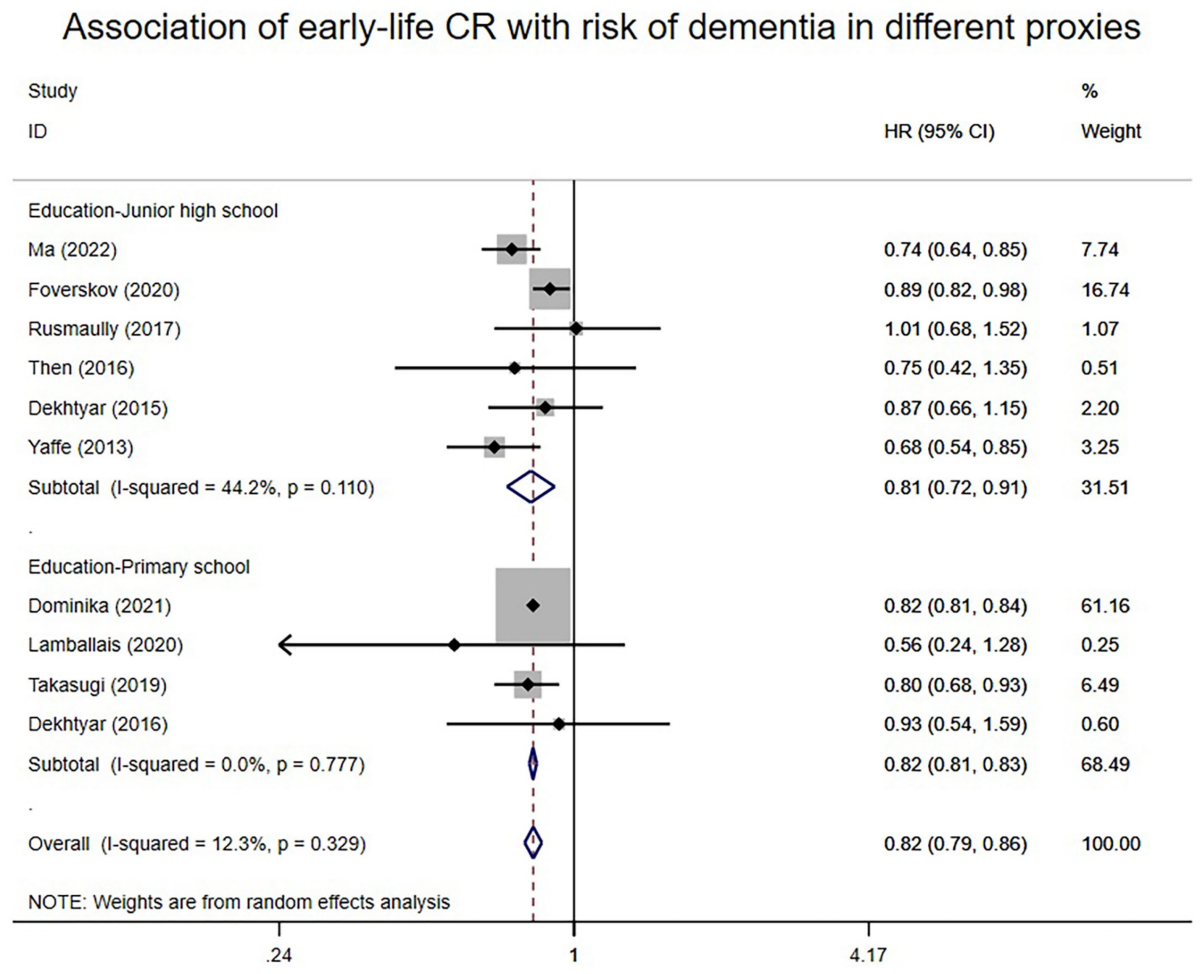
Association of early-life CR with risk of dementia in different proxies.

A total of 10 articles reported associations between proxies of early-life CR and the risk of dementia. A random-effects meta-analysis of early-life CR and the risk of dementia was conducted and is presented in Figure 2. Overall, individuals with high early-life CR had an 18% lower risk of dementia compared to those with low early-life CR (HR: 0.82; 95% CI: 0.79–0.86). Heterogeneity (I^2^) was low at 12.3%. Subgroup analysis of different CR proxy indicators in early-life revealed that high scores on these early-life CR proxies, including education up to junior high school (HR: 0.81; 95% CI: 0.72–0.91) and education up to primary school (HR: 0.82; 95% CI: 0.81–0.83), were associated with a relatively lower risk of dementia compared to low scores. I2 ranged from 0 to 44.2%.

#### 3.6.2 Middle-life CR and risk of dementia

**Figure 3:**
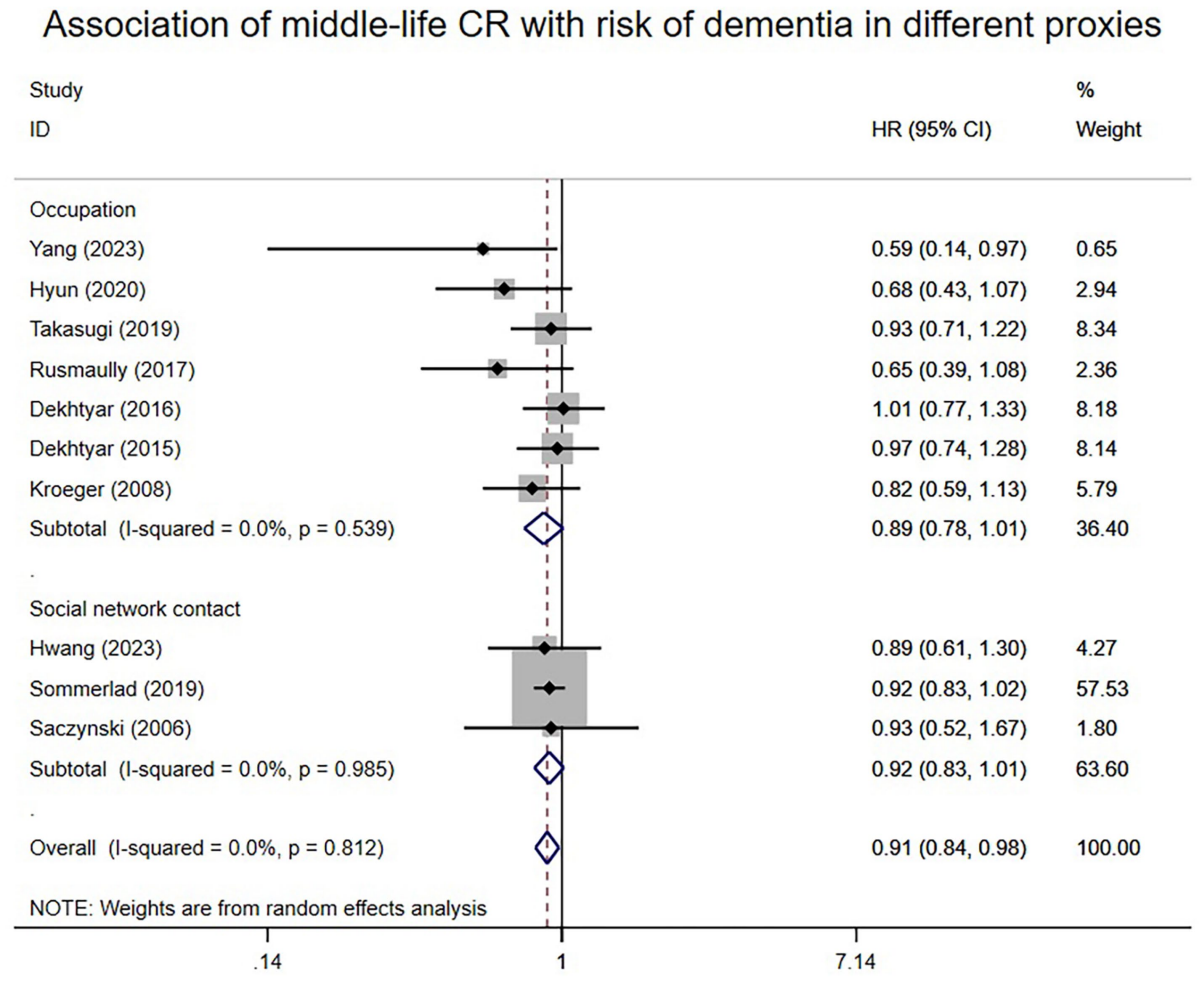
Association of middle-life CR with risk of dementia in different proxies.

A total of 10 studies reported associations between proxies of mid-life CR and the risk of dementia. A random-effects meta-analysis revealed that individuals with higher midlife CR tended to have a decreased risk of dementia (HR: 0.91; 95% CI: 0.84–0.98), as shown in Figure 3. I^2^ level was 0%. Subgroup analysis based on different mid-life CR proxies indicated that high social network contact (HR: 0.92; 95% CI: 0.83–1.01) and high occupational complexity (HR: 0.89; 95% CI: 0.78–1.01) may not be significantly associated with dementia risk.

#### 3.6.3 Late-life CR and risk of dementia

**Figure 4:**
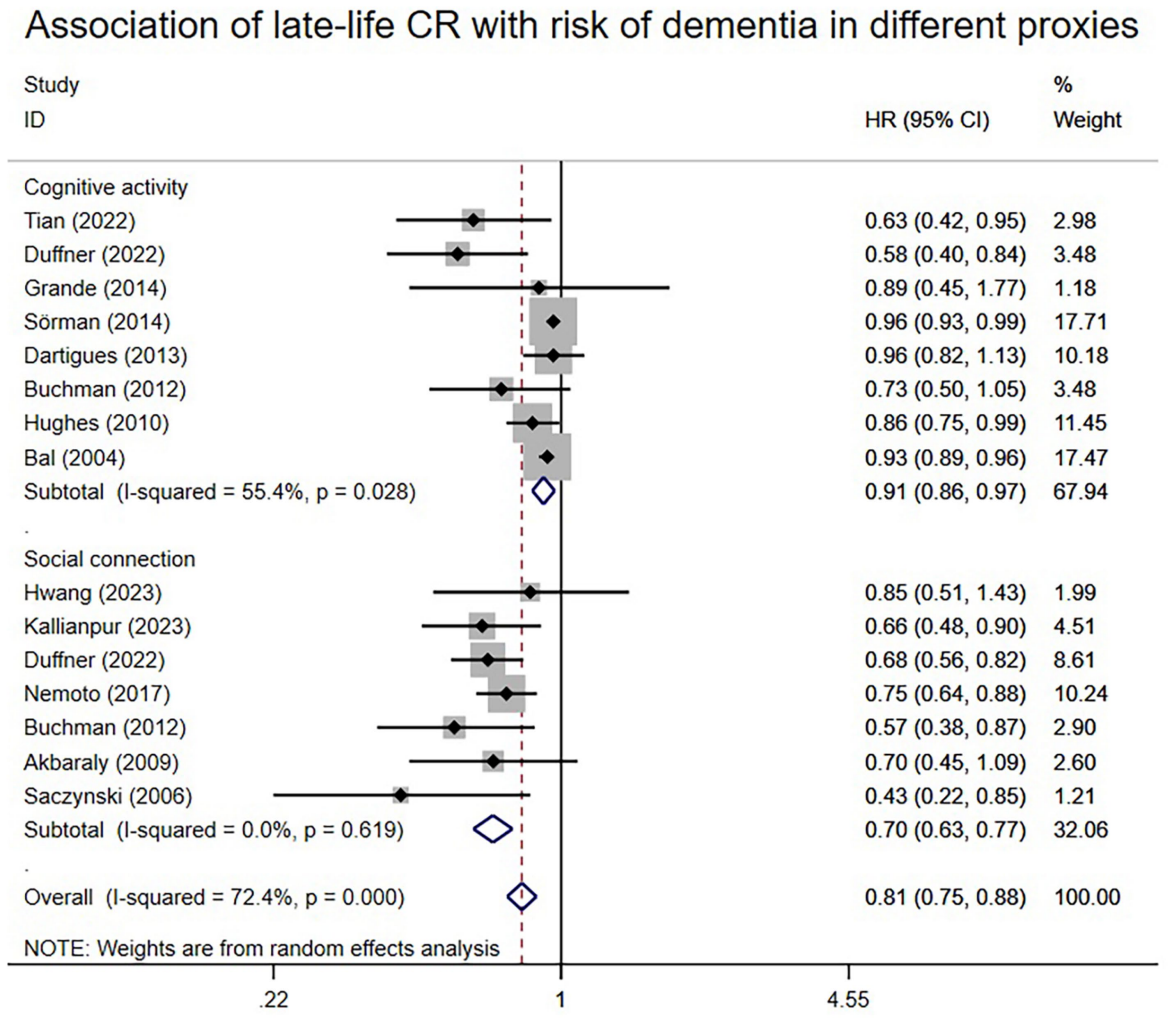
Association of late-life CR with risk of dementia in different proxies.

A total of 13 articles reported associations between proxies of late-life CR and the risk of dementia. Random-effects meta-analysis (Figure 4) illustrates these associations. The HR value was 0.81 (95% CI: 0.75–0.88), with an I^2^ level of 72.4%. All three proxies of cognitive activity and social connection exhibited statistically significant associations with the risk of dementia. The outcome of the meta-analysis indicated a subtotal HR of 0.91 (95% CI: 0.86–0.97) for cognitive activity and 0.70 (95% CI: 0.63–0.77) for social connection. I^2^ ranged from 0 to 55.4%, suggesting that CR proxies in late life may contribute to heterogeneity.

### 3.7 Sensitivity analyses

The results remained consistent even after switching from the random effects model to the fixed effects model in early life, middle life, and late-life (Supplementary Appendix D1). Similarly, consistency persisted when any single study was excluded from the meta-analyses of early-life, middle-life, and late-life (Supplementary Appendix D2). Subgroup analysis according to different proxies of CR also yielded consistent results (Figures 2–4). Although the Begg and Egger tests suggested potential publication bias in middle-life and late-life results, the trim-and-fill method did not add any studies, indicating the stability of the results (Supplementary Appendix B5).

## 4 Discussion

CR has been proposed as a compensatory mechanism to cope with age-related brain injury and to explain interindividual differences in the ability to maintain cognitive function in the presence of brain pathology (Xu et al., 2019). As individuals age, CR accumulates, and the proxies for each life stage may vary (Chapko et al., 2018). However, it remains unclear which life course stages and variables significantly influence dementia risk, with conflicting evidence supporting the association between CR and dementia risk (Takasugi et al., 2019; Wang et al., 2019; Zhang et al., 2021; Duffner et al., 2022). To address this gap in research, a meta-analysis was conducted examining the relationship between life-course CR and dementia risk.

### 4.1 CR proxy differences

In this systematic review and meta-analysis of 27 longitudinal studies, the association between CR and the risk of dementia over the life course (early, middle, and late life) was assessed.

#### 4.1.1 Early-life CR

In the assessment of individuals with high early CR, an 18% decreased risk of dementia was observed compared to those with poor early CR, as indicated by proxies for CR. Upon categorizing different proxies, varying levels of education were observed to correlate with differing degrees of dementia risk reduction. Educational attainment, the most commonly utilized CR proxy for early life, was significantly associated with a reduced risk of dementia later in life in the meta-analysis results. Further subgroup analysis of primary and junior high school education revealed a more pronounced effect of higher education levels on dementia risk among individuals with primary school education compared to those with junior high school education. This disparity may stem from the increasingly intricate neural network connections and heightened plasticity associated with higher education levels, enhancing the brain’s resilience to pathological damage. Based on these findings, emphasis should be placed on education. This conclusion is consistent with the studies by Seblova et al. (2021) and Xu et al. (2016) that found that each additional year of education beyond the baseline reduced the risk of dementia in later life. Thus, it is recommended to prioritize primary and secondary education, even if universal university education remains unattainable, as it plays a crucial role in promoting cognitive health later in life.

#### 4.1.2 Middle-life CR

Overall, mid-life CR accumulation was associated with a 9% decreased risk of dementia. Further subgroup analysis of CR proxies revealed varying effects on dementia risk. Specifically, a statistically significant association was observed between midlife occupational complexity and the risk of dementia. Occupational complexity serves as the most commonly used CR proxy indicator in midlife, providing the most accurate reflection of CR levels during this period. Consequently, its findings objectively depict the relationship between dementia risk and CR levels in middle age. The influence of occupational complexity on dementia onset may be realized through its complexity with people, things, and data. This suggests that different types of occupational complexity may have distinct associations with the risk of dementia. However, the limited research on the classification of occupational complexity necessitates further investigation. In a more thorough analysis, Kroeger et al. (2008) discovered that complexity with people or things, rather than data, was associated with a lower risk of dementia. Therefore, the various impacts of occupational complexity classification on dementia risk warrant further study.

The association between social contact and the risk of dementia in the current study was not significant. However, several studies (Hwang et al., 2023; Li et al., 2023) in older age found social contact to be significantly associated with the risk of dementia in later life. It was hypothesized that this absence of statistical significance was attributed to the limited literature on social contact during mid-life included in the current study. Sommerlad et al. (2019) found that although the association between social contact in mid-life and dementia risk was not statistically significant, more frequent social contact during midlife was associated with better subsequent cognitive trajectories. Therefore, considering that social isolation is a risk factor for dementia (Shen et al., 2022b), appropriate social contact in midlife remains necessary.

#### 4.1.3 Late-life CR

In comparison to early and middle life, there are significantly more studies conducted in late life. This trend may be attributed to the fact that longitudinal studies commencing in early or middle age require longer durations, and researchers often prefer to follow up in later life due to the higher likelihood of dementia development. Moreover, studies conducted in late life tend to have larger study populations, thereby enhancing the validity of their findings. Overall, individuals with high levels of late-life CR exhibited a 19% reduced incidence of dementia compared to those with low levels. Subgroup analysis of the proxies revealed that cognitive activity had a mitigating effect on the risk of dementia, with notably significant differences observed. Additionally, social connection may exert a relatively superior protective impact on dementia risk.

The results of a subgroup meta-analysis focusing on late-life CR and dementia risk underscore the significant association between social connection and dementia risk. This finding is consistent with similar conclusions drawn from other studies. For instance, Evans et al. (2019) conducted a meta-analysis investigating social isolation and cognitive function in later life, revealing that lower levels of social isolation, indicative of higher social engagement and connection, were linked to better cognitive function in later years. Notably, social loneliness and isolation are more prevalent among older adults compared to younger age groups. Shen et al. (2022a) further supported these findings, suggesting that social isolation might serve as an early indicator of heightened dementia risk, thereby advocating for early dementia prevention strategies. While there is currently no standardized international intervention (Fakoya et al., 2020), enhancing social networks and promoting increased social interaction among the elderly could represent a promising avenue for dementia prevention efforts.

Moreover, the current study revealed that engaging in cognitive activities such as reading, solving puzzles, and playing chess, and card games during later life was associated with a modest reduction in dementia risk. Notably, cognitive activity is classified as level A evidence for evidence-based prevention of Alzheimer’s disease (Yu et al., 2020). As individuals age, cognitively stimulating activities often decrease due to retirement and declines in physical function. However, maintaining cognitive activity in late life could help preserve the integrity of the brain’s white matter (Palta et al., 2021), potentially contributing to the observed association with reduced dementia risk.

### 4.2 Period differences

Overall, concerning different life stages, the protective effect of CR appears to be similar. Our findings indicate that CR in early and late life may confer a slightly higher protective effect against dementia risk compared to CR in middle life. This observation could be attributed to the fact that dementia typically manifests in later life, and the impact of CR proxies may be more pronounced when cognitive function has declined, especially compared to the cognitively normal midlife period. Alty et al. (2023) also suggested that CR interventions in old age may offer greater benefits, particularly for individuals with lower levels of education. This could be particularly encouraging for older males who may not have attained higher levels of education in their early years due to various reasons. Moreover, early-life CR accumulation demonstrated a more substantial protective effect, suggesting that while increasing CR at any stage is beneficial, accumulating CR earlier in life may offer greater risk reduction for dementia. This aligns with the findings of Wang et al. (2017), who proposed that higher exposure to CR proxies is associated with a reduced risk of dementia, highlighting the potential benefits of interventions initiated earlier in life.

### 4.3 Strengths and limitations of the study

In this review, the included studies were all longitudinal, providing a systematic and detailed understanding of the continuous developmental process and the laws of change. All eligible studies were published after 2004, with 70.4% of articles published in the last decade and 44.4% in the last 5 years, indicating the recent surge in researchers’ interest in the relationship between CR and dementia. The quality of the literature was assessed using internationally accepted standards, and all included studies were of medium to low-risk quality. Additionally, various sensitivity analyses were conducted to explore sources of heterogeneity, ensuring the stability and reliability of the experimental results.

Some limitations existed in the current study. For instance, although the quality of the literature was assessed using the NOS scale, since all studies measured CR in the form of questionnaires, the data reported by the subjects may have been somewhat biased from the actual situation and also led to some possible survivor bias. Additionally, various sensitivity analyses were employed to analyze the stability of the results, considering that the cohorts included in the studies were all healthy person cohorts, the effect of healthy cohort bias still needed to be considered. Studies came from different countries and regions, and some used different questionnaires, but the general direction was similar, and efforts were made to reduce such heterogeneity when screening the literature. Furthermore, Begg and Egger’s test indicated potential publication bias in studies in middle and late life. Nonetheless, the trim-and-fill method was conducted, and the results indicated stable conclusions. The I^2^ for late life was 72.4%, suggesting considerable heterogeneity, possibly because some studies reported results that were not expressed as HR and were therefore not included in the meta-analysis. To investigate the impact of late-life heterogeneity on the results of this study’s meta-analysis, several sensitivity analyses were conducted. These analyses demonstrated that the results of the meta-analysis remained unaffected, indicating the reliability of the conclusions.

Some CR proxies might not have been measured at every stage of the life course, potentially leading to missing indicators such as late-life occupation. This issue is also related to the insufficient number of existing studies, which could be updated if enough research on late-life occupation and dementia risk emerges in the future. Additionally, since most of the included studies initiated follow-up in late life, some data on the incidence of dementia in young and middle-aged individuals might have been inadequate. Limited by the amount of literature that met the criteria, this study focused on overall dementia risk. In the future, if possible, dementia could be typed, e.g., early-onset dementia and late-onset dementia, which may provide some recommendations and a basis for intervention in dementia prevention in the younger population. Considering the conceptual rigor of CR, this study did not include physical activity in the theoretical framework of the literature search. However, based on the available research (WHO, 2019), it is evident that exercise has many beneficial effects on brain health, helps reduce the risk of dementia, depression and stress, and plays a role in restoring and maintaining cognitive function and metabolic control, which is a strong protective factor against cognitive decline and dementia. Little is known about the mechanisms driving these effects, and future research could further investigate the relationship between CR, physical activity and dementia. In addition, due to the limitation of the number of available studies, meta-analysis of indicators determined by multiple factors (e.g., socioeconomic status) was not performed in this review. Referring to existing studies (Al Hazzouri et al., 2011), cumulative socioeconomic status also has a protective effect on dementia risk. Future studies could explore the relationship between indicators determined by multifaceted factors and dementia risk from the perspective of socioeconomic status. Unfortunately, given that little is known about the shared or separate underlying mechanisms in CR proxies, and given the differences in levels and variations in the effects of CR proxies on dementia risk, the present study cannot uncritically propose relevant interventions. This complexity is far from resolved and provides interesting directions for future work.

## 5 Conclusion

When synthesizing the findings from studies on CR and dementia risk across the life course, significant effects were observed for various CR proxies. These proxies included education in early life, occupation, social network contact in middle life, and cognitive activity and social connection in late life. The impact of CR proxies in early life and late life appeared to be more pronounced compared to middle life. Among these proxies, social connection emerged as potentially a effective approach for reducing dementia risk. However, given the impact of level versus change effects, further research is needed to determine which CR proxies to use at which stage to intervene in patients at risk for dementia.

## Author contributions

YL: Conceptualization, Data curation, Formal analysis, Investigation, Methodology, Project administration, Software, Validation, Visualization, Writing – original draft, Writing – review & editing. GL: Data curation, Methodology, Visualization, Writing – review & editing. LL: Conceptualization, Formal analysis, Methodology, Project administration, Supervision, Validation, Visualization, Writing – review & editing. YH: Data curation, Formal analysis, Investigation, Methodology, Software, Supervision, Validation, Visualization, Writing – review & editing. WG: Conceptualization, Formal analysis, Methodology, Project administration, Software, Supervision, Visualization, Writing – review & editing.
