# Supplementary material for: Cognitive reserve over the life course and risk of dementia: a systematic review and meta-analysis
Source: Front Aging Neurosci. 2024 Apr 12;16:1358992. doi: 10.3389/fnagi.2024.1358992 (PMC11047126; doi:10.3389/fnagi.2024.1358992)
Supplement: Supplementary file 1 [file Data_Sheet_1.docx]

Supplementary Material

# Appendix A. methods

a. Search algorithms.

Search strategy (concepts / block building approach)

| Overview databases and results  Date last searched: Jun 3, 2023 |
| --- |

Pubmed: 5449 records

1 "cognitive reserve*"[Title/Abstract] OR "brain reserve*"[Title/Abstract] OR "cognitive capacity"[Title/Abstract] OR "neural reserve"[Title/Abstract] OR "brain maintenance"[Title/Abstract] OR "cognitive resilience"[Title/Abstract] OR "brain resilience"[Title/Abstract] OR "education*"[Title/Abstract] OR "occupation*"[Title/Abstract] OR "leisure activit*"[Title/Abstract] (901,558)

2 "dementia"[Title/Abstract] OR "dement*"[Title/Abstract] OR "alzheim*"[Title/Abstract] (277,395)

3 "risk*"[Title/Abstract] OR "HR"[Title/Abstract] OR "hazard ratio"[Title/Abstract] (3,116,818)

4 1 and 2 and 3 (5,449)

Embase: 10773 records

#1. 'cognitive reserve'/exp OR 'cognitive reserve*':ab,ti OR 'brain reserve*':ab,ti OR 'cognitive capacity':ab,ti OR 'neural reserve':ab,ti OR 'brain maintenance':ab,ti OR 'cognitive resilience':ab,ti OR 'brain resilience':ab,ti OR education*:ab,ti OR occupation*:ab,ti OR 'leisure activit*':ab,ti (1,132,779)

#2. 'dementia'/exp OR dementia:ab,ti OR dement*:ab,ti OR alzheim*:ab,ti (508,296)

#3. 'risk'/exp OR risk*:ab,ti OR hr:ab,ti OR 'hazard ratio':ab,ti (5,320,196)

#4. #1 AND #2 AND #3 (10,773)

Web of science: 11816 records

#1 TS=("cognitive reserve*" OR "brain reserve*" OR "cognitive capacity" OR "neural reserve" OR "brain maintenance" OR "cognitive resilience" OR "brain resilience" OR education* OR occupation* OR leisure activit*) (2,716,149)

#2 TS=(dementia OR dement* OR Alzheim*) (497,768)

#3 TS=(risk* OR HR OR hazard ratio ) (5,504,687)

#4 #1 AND #2 AND #3 (11,816)

MEDLINE: 5203 records

S1 AB "cognitive reserve*" OR "brain reserve*" OR "cognitive capacity" OR "neural reserve" OR "brain maintenance" OR "cognitive resilience" OR "brain resilience" OR education* OR occupation* OR leisure activit* (732,377)

S2 AB dementia OR dement* OR Alzheim*  (243,947)

S3 AB risk* OR HR OR hazard ratio  (2,925,114)

S4 S1 AND S2 AND S3 (5,203)

b. Table A.1. List of excluded references after full-text screening

| Authors | Year | Title | Exclusion reason |
| --- | --- | --- | --- |
| Podewils, L. J., E. Guallar, L. H. Kuller, L. P. Fried, O. Lopez and C. G. Lyketsos | 2003 | Physical activity, APOE genotype, and dementia risk: Findings from the CHS cognition study | No full text |
| Fabrigoule, C. | 2002 | Do leisure activities protect against Alzheimer's disease? | Review |
| Ferrari, C., B. Nacmias, S. Bagnoli, I. Piaceri, G. Lombardi, S. Pradella, A. Tedde and S. Sorbi | 2014 | Imaging and cognitive reserve studies predict dementia in presymptomatic Alzheimer's disease subjects. | Review |
| Fratiglioni, L., A. Marseglia and S. Dekhtyar | 2020 | Ageing without dementia: can stimulating psychosocial and lifestyle experiences make a difference? | Review |
| Lövdén, M., L. Fratiglioni, M. M. Glymour, U. Lindenberger and E. M. Tucker-Drob | 2020 | Education and Cognitive Functioning Across the Life Span. | Review |
| Majoka, M. A. and C. Schimming | 2021 | Effect of Social Determinants of Health on Cognition and Risk of Alzheimer Disease and Related Dementias. | Review |
| Valenzuela, M. and P. S. Sachdev | 2009 | Harnessing brain and cognitive reserve for the prevention of dementia. | Review |
| Wilson, R. S. | 2005 | Mental challenge in the workplace and risk of dementia in old age: is there a connection? | Review |
| Almeida, P., A. Steptoe and D. Cadar | 2019 | MARKERS OF COGNITIVE RESERVE AND DEMENTIA INCIDENCE IN THE ENGLISH LONGITUDINAL STUDY OF AGEING. | Duplicate cohort |
| Almeida-Meza, P., A. P. Steptoe and D. Cadar | 2020 | ENGAGEMENT IN LEISURE ACTIVITIES AND DEMENTIA RISK IN THE ENGLISH LONGITUDINAL STUDY OF AGEING. | Duplicate cohort |
| Al-Najjar, J., I. Skoog, T. Hällström, S. Östling, P. Gudmundsson, L. Johansson, X. Guo and V. Sundh | 2015 | Leisure artistic and intellectual engagements and physical activity in midlife are associated with reduced risk of late-life dementia: A 38-year follow-up. | Duplicate cohort |
| Dekhtyar, S., A. Marseglia, W. Xu, A. Darin-Mattsson, H. X. Wang and L. Fratiglioni | 2018 | Similar dementia risk in APOE-ϵ4 carriers and non-carriers with high life-long cognitive reserve: A population-based cohort study. | Duplicate cohort |
| Dekhtyar, S., H. X. Wang, K. Scott, A. Goodman, I. Koupil and A. Herlitz | 2015 | A life-course study of cognitive reserve in dementia: Dementia incidence in inpatient registers and mmse test scores in a clinical study in Sweden. | Duplicate cohort |
| Epstein, E. F. | 2003 | Leisure activities and the risk of dementia. | Duplicate cohort |
| Feder, N. T., M. M. Bartley, J. I. Acosta, R. O. Roberts, D. S. Knopman, T. J. Christianson, V. S. Pankratz, M. M. Mielke, G. B. Stokin, V. J. Lowe, R. C. Petersen and Y. E. Geda | 2014 | Physical exercise and the outcome of incident dementia: The mayo clinic study of aging. | Duplicate cohort |
| Foubert-Samier, A., M. Le Goff, C. Helmer, K. Pérès, J. M. Orgogozo, P. Barberger-Gateau, H. Amieva and J. F. Dartigues | 2014 | Change in leisure and social activities and risk of dementia in elderly cohort. | Duplicate cohort |
| Gatz, M., P. Svedberg, N. L. Pedersen, J. A. Mortimer, S. Berg and B. Johansson | 2004 | Education and the risk of Alzheimer's disease: Findings from the study of dementia in Swedish twins (vol 56B, pg 292, 2001). | Duplicate cohort |
| Grande, G., I. Cova, L. Maggiore, S. Pomati, V. Cucumo, R. Ghiretti, M. Forcella, D. Galimberti, E. Scarpini, N. Vanacore, C. Mariani and F. Clerici | 2013 | High levels of participation in physical leisure activities protects MCI subjects against the risk of dementia. | Duplicate cohort |
| Hughes, T. F., C. C. H. Chang, J. Vanderbilt and M. Ganguli | 2010 | Engagement in reading and hobbies and incident dementia in the community: The MoVIES Project. | Duplicate cohort |
| Hyun, J., C. B. Hall, M. J. Katz, M. J. Sliwinski, C. Wang, A. Ezzati and R. B. Lipton | 2019 | THE ASSOCIATION BETWEEN MENTALLY CHALLENGING OCCUPATIONS AND INCIDENT DEMENTIA DIFFERS BETWEEN NON-HISPANIC WHITES AND AFRICAN-AMERICANS. | Duplicate cohort |
| Kroger, E., D. Laurin, R. Andel, J. Lindsay and R. Verreault | 2007 | Complexity of work and risk of dementia: The Canadian study of health and aging. | Duplicate cohort |
| Le Goff, M., C. Helmer, A. Foubert-Samier, P. Cowppli-Bony, C. Berr and J.-F. Dartigues | 2009 | [Activities in retired people and the risk of dementia]. | Duplicate cohort |
| Ott, A., C. T. van Rossum, F. van Harskamp, H. van de Mheen, A. Hofman and M. M. Breteler | 1999 | Education and the incidence of dementia in a large population-based study: the Rotterdam Study. | Duplicate cohort |
| Sanders, A. and J. Verghese | 2007 | Leisure activities and the risk of dementia in the elderly. | Duplicate cohort |
| Scarmeas, N., J. A. Luchsinger, N. Schupf, A. M. Brickman, S. Cosentino, M. X. Tang and Y. Stern | 2009 | Physical activity, diet, and risk of Alzheimer disease. | Duplicate cohort |
| Sommerlad | 2021 | Leisure Activity Participation and Risk of Dementia An 18-Year Follow-up of the Whitehall II Study (vol 95, pg e2803, 2020). | Duplicate cohort |
| Sommerlad, A., S. Sabia, A. Singh-Manoux, G. Lewis and G. Livingston | 2019 | ASSOCIATION OF SOCIAL NETWORK CONTACT WITH RISK OF DEMENTIA AND COGNITIVE DECLINE: 28-YEAR FOLLOW-UP OF THE WHITEHALL II COHORT STUDY. | Duplicate cohort |
| Cedar, D., C. Lassale, H. Davies, D. J. Llewellyn, D. Batty and A. Steptoe | 2018 | Individual and Area-Based Socioeconomic Factors Associated With Dementia Incidence in England Evidence From a 12-Year Follow-up in the English Longitudinal Study of Ageing. | Duplicate cohort |
| Takasugi, T., T. Tsuji, M. Hanazato, Y. Miyaguni, T. Ojima and K. Kondo | 2021 | Community-level educational attainment and dementia: a 6-year longitudinal multilevel study in Japan. | Duplicate cohort |
| Tyas, S., E. Hack and K. Riley | 2013 | Academic achievement in high school english courses and risk of Alzheimer's disease and dementia: Findings from the nun study. | Duplicate cohort |
| Deckers, K., D. Cadar, M. P. J. van Boxtel, F. R. J. Verhey, A. Steptoe and S. Köhler | 2019 | Modifiable Risk Factors Explain Socioeconomic Inequalities in Dementia Risk: Evidence from a Population-Based Prospective Cohort Study. | Duplicate cohort |
| Gilsanz, P., E. R. Mayeda, C. W. Eng, O. L. Meyer, M. Glymour and R. A. Whitmer | 2019 | CONCURRENCE BETWEEN OWN AND SPOUSAL EDUCATION AND DEMENTIA RISK IN A DIVERSE COHORT. | Duplicate cohort |
| Feder, N. T., M. M. Bartley, J. I. Acosta, R. O. Roberts, D. S. Knopman, T. J. Christianson, V. S. Pankratz, M. M. Mielke, G. B. Stokin, V. J. Lowe, R. C. Petersen and Y. E. Geda | 2014 | Physical exercise and the outcome of incident dementia: The mayo clinic study of aging. | Duplicate cohort |
| Dartiques, J. F., L. J. Launer, K. Andersen, A. Ott, L. Letenneur, P. Kragh-Sorensen, L. A. Amaducci, A. Lobo and J. M. Martinez-Lage | 1997 | The relation of education to the risk for incident Alzheimer's disease. | Duplicate cohort |
| Rodriguez, F. S., M. P. Aranda, D. A. Lloyd and W. A. Vega | 2018 | Racial and Ethnic Disparities in Dementia Risk Among Individuals With Low Education. | Duplicate cohort |
| van Oijen, M., F. J. de Jong, A. Hofman, P. J. Koudstaal and M. M. B. Breteler | 2007 | Subjective memory complaints, education, and risk of Alzheimer's disease. | Duplicate cohort |
| Vinkers, D. J., J. Gussekloo and R. G. Westendorp | 2003 | Leisure activities and the risk of dementia. | Duplicate cohort |
| Wang, H. X., S. Dekhtyar, L. Fratiglioni and A. Herlitz | 2015 | Childhood school performance, education, and occupational complexity: A life course study from the kungsholmen project. | Duplicate cohort |
| Xu, H., R. Yang, C. Dintica, X. Qi, R. Song, D. A. Bennett and W. Xu | 2020 | Association of lifespan cognitive reserve indicator with the risk of mild cognitive impairment and its progression to dementia. | Duplicate cohort |
| Xu, H., R. Yang, X. Qi, C. S. Dintica, R. Song, D. A. Bennett and W. Xu | 2019 | HIGH LIFESPAN COGNITIVE RESERVE IS ASSOCIATED WITH A REDUCED DEMENTIA RISK, INDEPENDENTLY OF BRAIN PATHOLOGIES. | Duplicate cohort |
| Deckers, K., D. Cadar, M. P. J. van Boxtel, F. R. J. Verhey, A. Steptoe and S. Köhler | 2019 | Modifiable Risk Factors Explain Socioeconomic Inequalities in Dementia Risk: Evidence from a Population-Based Prospective Cohort Study. | Duplicate cohort |
| Feder, N. T., M. M. Bartley, J. I. Acosta, R. O. Roberts, D. S. Knopman, T. J. Christianson, V. S. Pankratz, M. M. Mielke, G. B. Stokin, V. J. Lowe, R. C. Petersen and Y. E. Geda | 2014 | Physical exercise and the outcome of incident dementia: The mayo clinic study of aging. | Duplicate cohort |
| Yang, R., H. Xu, X. Qi, C. S. Dintica, D. A. Bennett and W. Xu | 2019 | HIGH LIFESPAN COGNITIVE RESERVE REDUCES THE RISK OF MILD COGNITIVE IMPAIRMENT AND DECELERATES ITS PROGRESSION TO DEMENTIA. | Duplicate cohort |
| Almeida-Meza, P., A. Steptoe and D. Cadar | 2021 | Is Engagement in Intellectual and Social Leisure Activities Protective Against Dementia Risk? Evidence from the English Longitudinal Study of Ageing. | Period unknown |
| Dufouil, C., E. Pereira, G. Chêne, M. M. Glymour, A. Alpérovitch, E. Saubusse, M. Risse-Fleury, B. Heuls, J. C. Salord, M. A. Brieu and F. Forette | 2013 | Older age at retirement is associated with decreased risk of dementia: Analysis of a health care insurance database of self-employed workers. | Period unknown |
| Fancourt, D., A. Steptoe and D. Cadar | 2020 | Community engagement and dementia risk: time-to-event analyses from a national cohort study. | Period unknown |
| Kitamura, K., Y. Watanabe, K. Kabasawa, A. Takahashi, T. Saito, R. Kobayashi, R. Takachi, R. Oshiki, S. Tsugane, M. Iki, A. Sasaki, O. Yamazaki, K. Watanabe and K. Nakamura | 2022 | Leisure-Time and Non-Leisure-Time Physical Activities are Dose-Dependently Associated With a Reduced Risk of Dementia in Community-Dwelling People Aged 40-74 Years: The Murakami Cohort Study. | Period unknown |
| Sanders, A. E., C. B. Hall, M. J. Katz and R. B. Lipton | 2012 | Non-native language use and risk of incident dementia in the elderly. | Period unknown |
| Shen, C., E. Rolls, W. Cheng, J. Kang, G. Dong, C. Xie, X.-M. Zhao, B. Sahakian and J. Feng | 2022 | Associations of Social Isolation and Loneliness With Later Dementia. | Period unknown |
| Sommerlad, A., S. Sabia, G. Livingston, M. Kivimäki, G. Lewis and A. Singh-Manoux | 2020 | Leisure activity participation and risk of dementia: An 18-year follow-up of the Whitehall II Study. | Period unknown |
| Summers, M., M. Valenzuela, J. Summers, K. Ritchie, T. Dickson, A. Robinson and J. Vickers | 2012 | The tasmanian healthy brain study (THBS): Does late-life education prevent age-related cognitive decline and dementia? | Period unknown |
| Then, F. S., T. Luck, K. Heser, A. Ernst, T. Posselt, B. Wiese, S. Mamone, C. Brettschneider, H.-H. König, S. Weyerer, J. Werle, E. Mösch, H. Bickel, A. Fuchs, M. Pentzek, W. Maier, M. Scherer, M. Wagner and S. G. Riedel-Heller | 2017 | Which types of mental work demands may be associated with reduced risk of dementia? | Period unknown |
| Xu, H., Yang, R., Dintica, C., Qi, X., Song, R., Bennett, D.A., Xu, W. | 2020 | Association of lifespan cognitive reserve indicator with the risk of mild cognitive impairment and its progression to dementia | Period unknown |
| Dekhtyar, S., Marseglia, A., Xu, W., Darin-Mattsson, A., Wang, H.-X., Fratiglioni, L. | 2019 | Genetic risk of dementia mitigated by cognitive reserve: A cohort study | Period unknown |
| Zabar, Y., M. Corrada, J. Fozard, P. Costa and C. Kawas | 1996 | Does frequent participation in cognitively demanding leisure activities reduce the risk of developing dementia? | Period unknown |
| Al Hazzouri, A. Z., M. N. Haan, J. D. Kalbfleisch, S. Galea, L. D. Lisabeth and A. E. Aiello | 2011 | Life-Course Socioeconomic Position and Incidence of Dementia and Cognitive Impairment Without Dementia in Older Mexican Americans: Results From the Sacramento Area Latino Study on Aging. | Period unknown |
| Zhang, Y., G. Natale and S. Clouston | 2021 | The Characteristics of Social Network Structure in Later Life in Relation to Incidence of Mild Cognitive Impairment and Conversion to Probable Dementia. | Lack of data |
| Ka Yan Lai, Chris Webster, Sarika Kumari, John E J Gallacher, Chinmoy Sarkar | 2022 | Association between individual-level socioeconomic position and incident dementia using UK Biobank data: a prospective study. | Lack of data |
| Allegri, R. F., F. E. Taragano, H. Krupitzki, C. M. Serrano, C. Dillon, D. Sarasola, M. Feldman, G. Tufró, M. Martelli and V. Sanchez | 2010 | Role of cognitive reserve in progression from mild cognitive impairment to dementia. | Lack of data |
| Fankhauser, S., Forstmeier, S., Maercker, A., Luppa, M., Luck, T., Riedel-Heller, S.G. | 2015 | Risk of dementia in older adults with low versus high occupation-based motivational processes: differential impact of frequency and proximity of social network | Lack of data |
| Tom, S.E., Phadke, M., Hubbard, R.A., Crane, P.K., Stern, Y., Larson, E.B. | 2020 | Association of Demographic and Early-Life Socioeconomic Factors by Birth Cohort With Dementia Incidence Among US Adults Born Between 1893 and 1949 | Lack of data |
| Shadlen, M.-F., D. Siscovick, A. L. Fitzpatrick, C. Dulberg, L. H. Kuller and S. Jackson | 2006 | Education, cognitive test scores, and black-white differences in dementia risk. | Lack of data |
| Kaup, A., E. Simonsick, T. Harris, S. Satterfield, A. Metti, H. Ayonayon, S. Rubin and K. Yaffe | 2013 | Limited literacy predicts dementia incidence among older adults. | Lack of data |
| Hendrie, H. C., V. Smith-Gamble, K. A. Lane, C. Purnell, D. O. Clark and S. Gao | 2018 | The Association of Early Life Factors and Declining Incidence Rates of Dementia in an Elderly Population of African Americans. | Lack of data |
| Letenneur, L., V. Gilleron, D. Commenges, C. Helmer, J. M. Orgogozo and J. F. Dartigues | 1999 | Are sex and educational level independent predictors of dementia and Alzheimer's disease? Incidence data from the PAQUID project. | Lack of data |
| Ojagbemi, A., T. Bello and O. Gureje | 2016 | Cognitive Reserve, Incident Dementia, and Associated Mortality in the Ibadan Study of Ageing. | Lack of data |
| Santabárbara, J., A. C. Gracía-Rebled, R. López-Antón, C. Tomás, E. Lobo, G. Marcos and A. Lobo | 2019 | The effect of occupation type on risk of Alzheimer's disease in men and women. | Lack of data |
| Takasugi, T., T. Tsuji, M. Hanazato, Y. Miyaguni, T. Ojima and K. Kondo | 2021 | Community-level educational attainment and dementia: a 6-year longitudinal multilevel study in Japan. | Lack of data |
| Appel, A. M., H. Brønnum-Hansen, A. H. Garde, Å. Hansen, K. Ishtiak-Ahmed, S. Islamoska, E. L. Mortensen, M. Osler and K. Nabe-Nielsen | 2022 | Socioeconomic Position and Late-Onset Dementia: A Nationwide Register-Based Study. | Lack of data |
| Rovio, S., B. Winblad, H. Soininen, J. Tuomilehto, A. Nissinen and M. Kivipelto | 2008 | Physical Activity Modifies Risk of Dementia and Alzheimer's Disease. | Lack of data |
| Donley, G. A. R., E. Lönnroos, T.-P. Tuomainen and J. Kauhanen | 2018 | Association of childhood stress with late-life dementia and Alzheimer's disease: the KIHD study. | Not CR enhancement factors |
| Gilsanz, P., C. P. Quesenberry, Jr., E. R. Mayeda, M. M. Glymour, S. T. Farias and R. A. Whitmer | 2019 | Stressors in Midlife and Risk of Dementia: The Role of Race and Education. | Not CR enhancement factors |
| Grande, G., D. L. Vetrano, I. Cova, S. Pomati, D. Mattavelli, L. Maggiore, V. Cucumo, R. Ghiretti, N. Vanacore, C. Mariani and D. Rizzuto | 2018 | Living Alone and Dementia Incidence: A Clinical-Based Study in People With Mild Cognitive Impairment. | Not CR enhancement factors |
| Goldberg, T. E., J. Choi, S. Lee, B. Gurland and D. P. Devanand | 2021 | Effects of restriction of activities and social isolation on risk of dementia in the community. | Not CR enhancement factors |
| Johansson, L., X. Guo, M. Norton, T. Hallstrom, M. Waern and I. Skoog | 2011 | Experiences of adverse life events and risk of dementia: A 38-year longitudinal population study of women. | Not CR enhancement factors |
| Wang, H.-X., M. Wahlberg, A. Karp, B. Winblad and L. Fratiglioni | 2012 | Psychosocial stress at work is associated with increased dementia risk in late life. | Not CR enhancement factors |
| Basu, R. | 2013 | Education and Dementia Risk: Results From the Aging Demographics and Memory Study. | Not longitudinal cohort |
| Petroianu, A., H. X. d. M. Capanema, M. M. Q. Silva and N. T. P. Braga | 2010 | Atividade física e mental no risco de demência em idosos. | Not longitudinal cohort |
| Russell, E. R., K. Stewart, D. F. Mackay, J. MacLean, J. P. Pell and W. Stewart | 2019 | Football's InfluencE on Lifelong health and Dementia risk (FIELD): protocol for a retrospective cohort study of former professional footballers. | Not longitudinal cohort |
| Zuelke, A. E., M. Luppa, S. Roehr, M. Weissenborn, A. Bauer, F.-A. Z. Samos, F. Kuehne, I. Zoellinger, J. Doehring, C. Brettschneider, A. Oey, D. Czock, T. Frese, J. Gensichen, W. E. Haefeli, W. Hoffmann, H. Kaduszkiewicz, H.-H. Koenig, J. R. Thyrian, B. Wiese and S. G. Riedel-Heller | 2021 | Association of mental demands in the workplace with cognitive function in older adults at increased risk for dementia. | Not longitudinal cohort |
| Juul Rasmussen I, Rasmussen KL, Thomassen JQ, Nordestgaard BG, Schnohr P, Tybjærg-Hansen A, Frikke-Schmidt R | 2022 | Physical activity in leisure time and at work and risk of dementia: A prospective cohort study of 117,616 individuals. | Not cognitive reserve |
| de Bruijn, R.F.A.G., Schrijvers, E.M.C., de Groot, K.A., Witteman, J.C.M., Hofman, A., Franco, O.H., Koudstaal, P.J. & Ikram, M.A. | 2013 | The association between physical activity and dementia in an elderly population: the Rotterdam Study. | Not cognitive reserve |
| Najar, J., Östling, S., Gudmundsson, P., Sundh, V., Johansson, L., Kern, S., Guo, X., Hällström, T. & Skoog, I. | 2019 | Cognitive and physical activity and dementia: A 44-year longitudinal population study of women. | Not cognitive reserve |
| Ogino, E., Manly, J.J., Schupf, N., Mayeux, R. & Gu, Y. | 2019 | Current and past leisure time physical activity in relation to risk of Alzheimer's disease in older adults. | Not cognitive reserve |
| Zhu J, Ge F, Zeng Y, Qu Y, Chen W, Yang H, Yang L, Fang F, Song H | 2022 | Physical and Mental Activity, Disease Susceptibility, and Risk of Dementia: A Prospective Cohort Study Based on UK Biobank. | Not cognitive reserve |
| Sundström, A., D. E. Sörman, P. Hansson, J. K. Ljungberg and R. Adolfsson | 2020 | Mental Demands at Work and Risk of Dementia. | Not cognitive reserve |
| Tan, X., A. Lebedeva, T. Åkerstedt and H.-X. Wang | 2022 | Sleep mediates the association between stress at work and incident dementia: study from the Survey of Health, Ageing and Retirement in Europe. | Not cognitive reserve |
| Kunutsor, S. K., J. A. Laukkanen, J. Kauhanen and P. Willeit | 2021 | Physical activity may not be associated with long-term risk of dementia and Alzheimer's disease. | Not cognitive reserve |
| Garcia, J., K. Erickson, C. Raji, O. Lopez, A. Newman, C. Rosano and L. Kuller | 2011 | Physical activity is predictive of dementia but not mortality. | Not cognitive reserve |
| Ihira, H., N. Sawada, M. Inoue, N. Yasuda, K. Yamagishi, H. Charvat, M. Iwasaki and S. Tsugane | 2022 | Association Between Physical Activity and Risk of Disabling Dementia in Japan. | Not cognitive reserve |
| Almeida, O. P., B. B. Yeap, H. Alfonso, G. J. Hankey, L. Flicker and P. E. Norman | 2012 | Older men who use computers have lower risk of dementia. | Not cognitive reserve |
| Arafa, A., E. S. Eshak, K. Shirai, D. Cadar, H. Iso, T. Tsuji, S. Kanamori and K. Kondo | 2021 | Impact of various intensities and frequencies of non-occupational physical activity on the risk of dementia among physically independent older adults: the Japan Gerontological Evaluation Study. | Not cognitive reserve |
| Bokenberger, K., A. Sjölander, A. K. Dahl Aslan, I. K. Karlsson, T. Åkerstedt and N. L. Pedersen | 2017 | Midlife shift work and risk of incident dementia. | Not cognitive reserve |
| Cadar, D., R. A. Hackett, M. Mischie, D. J. Llewellyn, G. D. Batty and A. Steptoe | 2017 | Association of physical activity as a distinctive feature of clustering of lifestyle behaviours with dementia risk: Evidence from the English Longitudinal Study of Ageing. | Not cognitive reserve |
| Camozzato, A., C. Godinho, J. Varela, C. Kohler, J. Rinaldi and M. L. Chaves | 2015 | The complex role of having confidant on the development of Alzheimer's disease in a community-based cohort of older people in Brazil. | Not cognitive reserve |
| Dhana, K., D. A. Evans, K. B. Rajan, D. A. Bennett and M. C. Morris | 2019 | IMPACT OF HEALTHY LIFESTYLE FACTORS ON THE RISK OF ALZHEIMER'S DEMENTIA: FINDINGS FROM TWO PROSPECTIVE COHORT STUDIES. | Not cognitive reserve |
| Verghese, J., Lipton, R.B., Katz, M.J., Hall, C.B., Derby, C.A., Kuslansky, G., Ambrose, A.F., Sliwinski, M. & Buschke, H. | 2003 | Leisure activities and the risk of dementia in the elderly. | Not cognitive reserve |
| Krell-Roesch, J., Feder, N.T., Roberts, R.O., Mielke, M.M., Christianson, T.J., Knopman, D.S., Petersen, R.C. & Geda, Y.E. | 2018 | Leisure-Time Physical Activity and the Risk of Incident Dementia: The Mayo Clinic Study of Aging. | Not cognitive reserve |
| Kishimoto, H., Ohara, T., Hata, J., Ninomiya, T., Yoshida, D., Mukai, N., Nagata, M., Ikeda, F., Fukuhara, M., Kumagai, S., Kanba, S., Kitazono, T. & Kiyohara, Y. | 2016 | The long-term association between physical activity and risk of dementia in the community: the Hisayama Study. | Not cognitive reserve |
| Forstmeier, S., Maercker, A., Maier, W., van den Bussche, H., Riedel-Heller, S., Kaduszkiewicz, H., Pentzek, M., Weyerer, S., Bickel, H., Tebarth, F., Luppa, M., Wollny, A., Wiese, B., Wagner, M. | 2012 | Motivational reserve: motivation-related occupational abilities and risk of mild cognitive impairment and Alzheimer disease. | Not cognitive reserve |
| Huang, A. R., K. L. Strombotne, E. M. Horner and S. J. Lapham | 2018 | Adolescent Cognitive Aptitudes and Later-in-Life Alzheimer Disease and Related Disorders. | Not cognitive reserve |
| Wu, W., Ding, D., Zhao, Q., Wang, R., Liang, X., Xiao, Z., Luo, J., Guo, Q. & Hong, Z. | 2020 | Medium-to-High Late-Life Physical Activity Is Associated with Lower Risk of Incident Dementia: The Shanghai Aging Study. | Not cognitive reserve |
| Ravaglia, G., Forti, P., Lucicesare, A., Pisacane, N., Rietti, E., Bianchin, M. & Dalmonte, E. | 2008 | Physical activity and dementia risk in the elderly - Findings from a prospective Italian study. | Not cognitive reserve |
| Najar, J., J. A. Aakre, M. Vassilaki, H. Wetterberg, L. Rydén, A. Zettergren, I. Skoog, C. R. Jack, D. S. Knopman, R. C. Petersen, S. Kern and M. M. Mielke | 2021 | Sex Difference in the Relation Between Marital Status and Dementia Risk in Two Population-Based Cohorts. | Not cognitive reserve |
| Palta, P., K. P. Gabriel, A. Kumar, A. R. Sharrett, K. R. Evenson, R. F. Gottesman, T. Mosley, G. M. Heiss and K. Diaz | 2021 | Sedentary behavior in mid-life and risk of change in global cognitive function and incident dementia: The atherosclerosis risk in communities neurocognitive study (Aric-Ncs). | Not cognitive reserve |
| Liu, Y., Mitsuhashi, T., Yamakawa, M., Sasai, M., Tsuda, T., Doi, H. & Hamada, J. | 2019 | Physical activity and incident dementia in older Japanese adults: The Okayama study. | Not cognitive reserve |
| Pan, K. Y., W. Xu, F. Mangialasche, G. Grande, L. Fratiglioni and H. X. Wang | 2019 | PASSIVE JOB AND DEMENTIA RISK IN OLDER ADULTS: THE ROLE OF APOLIPOPROTEIN E. | Not cognitive reserve |
| Taaffe, D.R., Irie, F., Masaki, K.H., Abbott, R.D., Petrovitch, H., Ross, G.W. & White, L.R. | 2008 | Physical activity, physical function, and incident dementia in elderly men: the Honolulu-Asia Aging Study. | Not cognitive reserve |
| Power, M. C., A. E. Murphy, K. Z. Gianattasio, Y. I. Zhang, R. L. Walker, P. K. Crane, E. B. Larson, L. E. Gibbons, R. G. Kumar and K. Dams-O'Connor | 2021 | Association of Military Employment With Late-Life Cognitive Decline and Dementia: A Population-Based Prospective Cohort Study. | Not cognitive reserve |
| Romero, J., A. Medel, F. Bermejo-Pareja, R. Trincado, A. Sanchez, F. Sierra, A. Herrero, A. V. Galende, J. Benito-Leon and S. Vega | 2013 | Physical activity and risk of alzheimer disease: Data from the nedices cohort. | Not cognitive reserve |
| Palta, P., Sharrett, A.R., Deal, J.A., Evenson, K.R., Gabriel, K.P., Folsom, A.R., Gross, A.L., Windham, B.G., Knopman, D., Mosley, T.H. & Heiss, G. | 2019 | Leisure-time physical activity sustained since midlife and preservation of cognitive function: The Atherosclerosis Risk in Communities Study. | Not cognitive reserve |
| Sanders, A., C. Hall, M. Katz and R. Lipton | 2011 | Bilingualism and incident dementia risk: Results from the einstein aging study. | Not cognitive reserve |
| Hansson, O., Svensson, M., Gustavsson, A.M., Andersson, E., Yang, Y., Nägga, K., Hållmarker, U., James, S. & Deierborg, T. | 2019 | Midlife physical activity is associated with lower incidence of vascular dementia but not Alzheimer's disease. | Not cognitive reserve |
| Simons, L. A., J. Simons, J. McCallum and Y. Friedlander | 2006 | Lifestyle factors and risk of dementia: Dubbo Study of the elderly. | Not cognitive reserve |
| Solomon, A., R. Wang, S. Sindi, C. Qiu, T. Ngandu, M. A. Ikram, B. Winblad, L. Fratiglioni and M. Kivipelto | 2017 | Risk score for prediction of dementia risk in 10 years among older adults: A nordic populationbased study with three cohorts. | Not cognitive reserve |
| Zotcheva, E., Bergh, S., Selbæk, G., Krokstad, S., Håberg, A.K., Strand, B.H. & Ernstsen, L. | 2018 | Midlife Physical Activity, Psychological Distress, and Dementia Risk: The HUNT Study. | Not cognitive reserve |
| Sundström, A., O. Westerlund and E. Kotyrlo | 2016 | Marital status and risk of dementia: a nationwide population-based prospective study from Sweden. | Not cognitive reserve |
| Sundström, A., O. Westerlund, H. Mousavi-Nasab, R. Adolfsson and L. G. Nilsson | 2013 | Relationship between marital and parental status and risk of dementia and Alzheimer's disease. | Not cognitive reserve |
| Tani, Y., M. Hanazato, T. Fujiwara, N. Suzuki and K. Kondo | 2021 | Neighborhood Sidewalk Environment and Incidence of Dementia in Older Japanese Adults. | Not cognitive reserve |
| Wang, F., J. A. Mortimer, D. Ding, J. Luo, Q. Zhao, X. Liang, W. Wu, L. Zheng, Q. Guo, A. R. Borenstein and Z. Hong | 2019 | Smaller Head Circumference Combined with Lower Education Predicts High Risk of Incident Dementia: The Shanghai Aging Study. | Not cognitive reserve |
| Wang, L., E. B. Larson, J. D. Bowen and G. van Belle | 2006 | Performance-based physical function and future dementia in older people. | Not cognitive reserve |
| Yang, J. J., L. M. Keohane, X. Pan, R. Qu, X.-O. Shu, L. P. Lipworth, K. Braun, M. D. Steinwandel, Q. Dai, M. Shrubsole, W. Zheng, W. J. Blot and D. Yu | 2022 | Association of Healthy Lifestyles with Risk of Alzheimer Disease and Related Dementias in Low-Income Black and White Americans. | Not cognitive reserve |
| Yun, S.-H., S.-H. Jo, H.-S. Jung, B.-H. Koo and H.-G. Kim | 2020 | Characteristics of Individuals Who Converted to Dementia during a 5-Year Follow-Up. | Not cognitive reserve |
| Adam, S., E. Bonsang, C. Grotz and S. Perelman | 2013 | Occupational activity and cognitive reserve: Implications in terms of prevention of cognitive aging and Alzheimer's disease. | No risk of dementia |
| Arora, K., L. Xu and D. Bhagianadh | 2021 | Dementia and Cognitive Decline in Older Adulthood: Are Agricultural Workers at Greater Risk? | No risk of dementia |
| Palmer, K., H. Inskip, C. Martyn and D. Coggon | 1998 | Dementia and occupational exposure to organic solvents. | No risk of dementia |
| Bermejo-Pareja, F., A. Medel, J. Benito-León, P. Siebel and A. Herrero-San Martín | 2012 | Physical activity is a protective factor for dementia. Data from NEDICES cohort. | No risk of dementia |
| Bruandet, A., F. Richard, S. Bombois, C. A. Maurage, I. Masse, P. Amouyel and F. Pasquier | 2008 | Cognitive decline and survival in Alzheimer's disease according to education level. | No risk of dementia |
| Chen, Y.-C., M. Putnam, Y. S. Lee and N. Morrow-Howell | 2019 | Activity Patterns and Health Outcomes in Later Life: The Role of Nature of Engagement. | No risk of dementia |
| Fernandez-Matarrubia, M., L. Goni, T. Rognoni, C. Razquin, C. Ignacio Fernandez-Lazaro, M. Bes-Rastrollo, M. Angel Martinez-Gonzalez and E. Toledo | 2021 | An Active Lifestyle Is Associated with Better Cognitive Function Over Time in APOE epsilon 4 Non-Carriers. | No risk of dementia |
| Kesavayuth, D., Y. Liang and V. Zikos | 2018 | An active lifestyle and cognitive function: Evidence from China. | No risk of dementia |
| Fratiglioni, L., M. Viitanen, E. Von Strauss and B. Winblad | 1996 | The risk of late onset Alzheimer's disease in relation to gender and education. Incidence data from the Kungsholmen Project, Stockholm. | No risk of dementia |
| Gross, A. L., H. Lu, L. Meoni, J. J. Gallo, J. A. Schrack and A. R. Sharrett | 2017 | Physical Activity in Midlife is not Associated with Cognitive Health in Later Life Among Cognitively Normal Older Adults. | No risk of dementia |
| Almeida-Meza, P., Steptoe, A., Cadar, D. | 2021 | Markers of cognitive reserve and dementia incidence in the English Longitudinal Study of Ageing | No risk of dementia |
| Crooks, V.C., Lubben, J., Petitti, D.B., Little, D., Chiu, V. | 2008 | Social network, cognitive function, and dementia incidence among elderly women | No risk of dementia |
| Hakansson, K., E. L. Helkala, H. Soininen, A. Nissinen, A. Mohammed, B. Winblad and M. Kivipelto | 2011 | Perceived marital problems in midlife are associated with cognitive health in later life. | No risk of dementia |
| Ho, S. C., J. Woo, A. Sham, S. G. Chan and A. L. M. Yu | 2001 | A 3-year follow-up study of social, lifestyle and health predictors of cognitive impairment in a Chinese older cohort. | No risk of dementia |
| Hughes, T. F., Z. Sun, C.-C. H. Chang and M. Ganguli | 2018 | Change in Engagement in Cognitive Activity and Risk for Mild Cognitive Impairment in a Cohort of Older Adults: The Monongahela-Youghiogheny Healthy Aging Team (MYHAT) Study. | No risk of dementia |
| Hyun, J., M. J. Katz, R. B. Lipton and M. J. Sliwinski | 2021 | Mentally Challenging Occupations Are Associated With More Rapid Cognitive Decline at Later Stages of Cognitive Aging. | No risk of dementia |
| Ihira, H., N. Sawada, M. Inoue, N. Yasuda, K. Yamagishi, H. Charvat, M. Iwasaki and S. Tsugane | 2022 | Association Between Physical Activity and Risk of Disabling Dementia in Japan. | No risk of dementia |
| Iso-Markku, P., J. Kaprio, N. Lindgren, J. O. Rinne and E. Vuoksimaa | 2021 | Middle-age dementia risk scores and old-age cognition: a quasi-experimental population-based twin study with over 20-year follow-up. | No risk of dementia |
| Jin, X., W. He, Y. Zhang, E. Gong, Z. Niu, J. Ji, Y. Li, Y. Zeng and L. L. Yan | 2021 | Association of APOE epsilon 4 genotype and lifestyle with cognitive function among Chinese adults aged 80 years and older: A cross-sectional study. | No risk of dementia |
| Kaplan, G. A., G. Turrell, J. W. Lynch, S. A. Everson, E. L. Helkala and J. T. Salonen | 2001 | Childhood socioeconomic position and cognitive function in adulthood. | No risk of dementia |
| Krell-Roesch, J., P. Vemuri, A. Pink, R. O. Roberts, G. B. Stokin, M. M. Mielke, T. J. H. Christianson, D. S. Knopman, R. C. Petersen, W. K. Kremers and Y. E. Geda | 2017 | Association Between Mentally Stimulating Activities in Late Life and the Outcome of Incident Mild Cognitive Impairment, With an Analysis of the APOE ε4 Genotype. | No risk of dementia |
| Kurita, S., K. Tsutsumimoto, T. Doi, S. Nakakubo, M. Kim, H. Ishii and H. Shimada | 2020 | Association of physical and/or cognitive activity with cognitive impairment in older adults. | No risk of dementia |
| Marioni, R. E., A. Van Den Hout, M. J. Valenzuela, C. Brayne and F. E. Matthews | 2012 | Active cognitive lifestyle associates with cognitive recovery and a reduced risk of cognitive decline. | No risk of dementia |
| Mortimer, J. A., A. R. Borenstein, A. Mbah, P. K. Crane and E. B. Larson | 2017 | Role of reserve in reducing dementia: Comparison of two large clinicopathologic studies. | No risk of dementia |
| Munoz, D. G., G. R. Ganapathy, M. Eliasziw and V. Hachinski | 2000 | Educational attainment and socioeconomic status of patients with autopsy-confirmed Alzheimer disease. | No risk of dementia |
| Petersen, J. D., S. Wehberg, A. Packness, N. H. Svensson, N. Hyldig, S. Raunsgaard, M. K. Andersen, J. Ryg, S. W. Mercer, J. Sondergaard and F. B. Waldorff | 2021 | Association of Socioeconomic Status With Dementia Diagnosis Among Older Adults in Denmark. | No risk of dementia |
| Roberts, J. and M. Maxfield | 2016 | THE IMPACT OF ALZHEIMER'S DISEASE RISK REDUCTION EDUCATION ON DEMENTIA WORRY. | No risk of dementia |
| Roberts, R. | 2011 | MCI incidence, progression to dementia, and reversion to normal in a population-based cohort: The mayo clinic study of aging. | No risk of dementia |
| Schmand, B., J. Lindeboom, C. Hooijer and C. Jonker | 1995 | Relation between education and dementia: the role of test bias revisited. | No risk of dementia |
| Shakersain, B., D. Rizzuto, H.-X. Wang, G. Faxén-Irving, F. Prinelli, L. Fratiglioni and W. Xu | 2018 | An Active Lifestyle Reinforces the Effect of a Healthy Diet on Cognitive Function: A Population-Based Longitudinal Study. | No risk of dementia |
| Strand, B. H., E. M. Langballe, T. A. Rosness, A. L. M. Bergem, K. Engedal, P. Nafstad, G. S. Tell, H. Ormstad, K. Tambs and E. Bjertness | 2014 | Age, education and dementia related deaths. The Norwegian Counties Study and The Cohort of Norway. | No risk of dementia |
| Sumic, A., Y. L. Michael, N. E. Carlson, D. B. Howieson and J. A. Kaye | 2007 | Physical activity and the risk of dementia in oldest old. | No risk of dementia |
| Vemuri, P., T. G. Lesnick, S. A. Przybelski, M. Machulda, D. S. Knopman, M. M. Mielke, R. O. Roberts, Y. E. Geda, W. A. Rocca, R. C. Petersen and C. R. Jack | 2014 | Association of Lifetime Intellectual Enrichment With Cognitive Decline in the Older Population. | No risk of dementia |
| Wu, W., D. Ding, Q. Zhao, Z. Xiao, M. Ganguli, M. Haan, M. F. Lima-Costa, E. Castro-Costa, T. P. Ng, O. Gureje, N. Scarmeas, H. Brodaty, R. Lipton, M. Katz, D. Lipnicki and P. Sachdev | 2021 | Dose-response association of the duration and volume of physical activity with incident dementia: A cosmic collaborative cohort study. | No risk of dementia |
| Xu, W., B. Shakersain, D. Rizzuto, H. X. Wang, G. F. Irving and L. Fratiglioni | 2018 | AN ACTIVE LIFESTYLE REINFORCES THE PROTECTIVE EFFECT OF A HEALTHY DIET ON COGNITIVE FUNCTION. | No risk of dementia |
| Kaup, A.R., Simonsick, E.M., Harris, T.B., Satterfield, S., Metti, A.L., Ayonayon, H.N., Rubin, S.M., Yaffe, K. | 2014 | Older adults with limited literacy are at increased risk for likely dementia. | No risk of dementia |
| Andel, R., M. Crowe, N. L. Pedersen, L. Fratiglioni, B. Johansson and M. Gatz | 2008 | Physical exercise at midlife and risk of dementia three decades later: a population-based study of Swedish twins. | Outcome not HRs |
| Andel, R., M. Crowe, N. L. Pedersen, J. Mortimer, E. Crimmins, B. Johansson and M. Gatz | 2005 | Complexity of work and risk of Alzheimer's disease: a population-based study of Swedish twins. | Outcome not HRs |
| Anderson, E. L., L. D. Howe, K. H. Wade, Y. Ben-Shlomo, W. D. Hill, I. J. Deary, E. C. Sanderson, J. Zheng, R. Korologou-Linden, E. Stergiakouli, G. Davey Smith, N. M. Davies and G. Hemani | 2020 | Education, intelligence and Alzheimer's disease: evidence from a multivariable two-sample Mendelian randomization study. | Outcome not HRs |
| Andersson, M. A., S. K. Gadarian and R. Almeling | 2017 | Does educational attainment shape reactions to genetic risk for Alzheimer's disease? Results from a national survey experiment. | Outcome not HRs |
| Artero, S., M. L. Ancelin, F. Portet, A. Dupuy, C. Berr, J. F. Dartigues, C. Tzourio, O. Rouaud, M. Poncet, F. Pasquier, S. Auriacombe, J. Touchon and K. Ritchie | 2008 | Risk profiles for mild cognitive impairment and progression to dementia are gender specific. | Outcome not HRs |
| Astell-Burt, T. and X. Feng | 2018 | Is the risk of developing Alzheimer's disease really higher in rural areas? A multilevel longitudinal study of 261,669 Australians aged 45 years and older tracked over 11 years. | Outcome not HRs |
| Barcelos-Ferreira, R., M. Lopes and C. Bottino | 2013 | Physical activity associated with lower prevalence of dementia. | Outcome not HRs |
| Bezerra, A. B. C., E. S. F. Coutinho, M. L. Barca, K. Engedal, E. Engelhardt and J. Laks | 2012 | School attainment in childhood is an independent risk factor of dementia in late life: results from a Brazilian sample. | Outcome not HRs |
| Bickel, H. and A. Kurz | 2009 | Education, occupation, and dementia: the Bavarian school sisters study. | Outcome not HRs |
| Bonaiuto, S., W. A. Rocca, A. Lippi, E. Giannandrea, M. Mele, F. Cavarzeran and L. Amaducci | 1995 | Education and occupation as risk factors for dementia: a population-based case-control study. | Outcome not HRs |
| Borenstein, A., J. Mortimer, D. Ding, Q. Zhao, S. Chu, Q. Guo, C. DeCarli and Z. Hong | 2012 | Minutes of walking per dayat age 50 is associated with dementia risk, and this association is partially mediated by whole brain volume in women: Scobhi-P. | Outcome not HRs |
| Carlson, M. C., M. J. Helms, D. C. Steffens, J. R. Burke, G. G. Potter and B. L. Plassman | 2008 | Midlife activity predicts risk of dementia in older male twin pairs. | Outcome not HRs |
| Chen, J. H., Y. C. Chen, S. Y. Yang, S. Y. Chen, P. K. Yip, T. F. Chen, Y. Sun, L. L. Wen and Y. M. Chu | 2012 | Leisure activities, apoe-ε4 status and Alzheimer's disease. | Outcome not HRs |
| Cobb, J. L., P. A. Wolf, R. Au, R. White and R. B. D'Agostino | 1995 | The effect of education on the incidence of dementia and Alzheimer's disease in the Framingham Study. | Outcome not HRs |
| Contador, I., F. Bermejo-Pareja, V. Puertas-Martin and J. Benito-Leon | 2015 | Childhood and Adulthood Rural Residence Increases the Risk of Dementia: NEDICES Study. | Outcome not HRs |
| Crowe, M., R. Andel, N. L. Pedersen, B. Johansson and M. Gatz | 2003 | Does participation in leisure activities lead to reduced risk of Alzheimer's disease? A prospective study of Swedish twins. | Outcome not HRs |
| Darwish, H., N. Farran, S. Assaad and M. Chaaya | 2018 | Cognitive Reserve Factors in a Developing Country: Education and Occupational Attainment Lower the Risk of Dementia in a Sample of Lebanese Older Adults. | Outcome not HRs |
| De Ronchi, D., L. Fratiglioni, P. Rucci, A. Paternicò, S. Graziani and E. Dalmonte | 1998 | The effect of education on dementia occurrence in an Italian population with middle to high socioeconomic status. | Outcome not HRs |
| Evans, D. A., L. E. Hebert, L. A. Beckett, P. A. Scherr, M. S. Albert, M. J. Chown, D. M. Pilgrim and J. O. Taylor | 1997 | Education and other measures of socioeconomic status and risk of incident Alzheimer disease in a defined population of older persons. | Outcome not HRs |
| Fabrigoule, C., L. Letenneur, J. F. Dartigues, M. Zarrouk, D. Commenges and P. Barberger-Gateau | 1995 | Social and leisure activities and risk of dementia: a prospective longitudinal study. | Outcome not HRs |
| Fan, L.-Y., Y. Sun, H.-J. Lee, S.-C. Yang, T.-F. Chen, K.-N. Lin, C.-C. Lin, P.-N. Wang, L.-Y. Tang and M.-J. Chiu | 2015 | Marital Status, Lifestyle and Dementia: A Nationwide Survey in Taiwan. | Outcome not HRs |
| Floud, S., A. Balkwill, S. Sweetland, A. Brown, E. M. Reus, A. Hofman, D. Blacker, M. Kivimaki, J. Green, R. Peto, G. K. Reeves and V. Beral | 2021 | Cognitive and social activities and long-term dementia risk: the prospective UK Million Women Study. | Outcome not HRs |
| Fratiglioni, L., H. X. Wang, K. Ericsson, M. Maytan and B. Winblad | 2000 | Influence of social network on occurrence of dementia: a community-based longitudinal study. | Outcome not HRs |
| Gatz, M., J. A. Mortimer, L. Fratiglioni, B. Johansson, S. Berg, R. Andel, M. Crowe, A. Fiske, C. A. Reynolds and N. L. Pedersen | 2007 | Accounting for the relationship between low education and dementia: a twin study. | Outcome not HRs |
| Gatz, M., P. Svedberg, N. L. Pedersen, J. A. Mortimer, S. Berg and B. Johansson | 2001 | Education and the risk of Alzheimer's disease: findings from the study of dementia in Swedish twins. | Outcome not HRs |
| Geerlings, M. I., B. Schmand, C. Jonker, J. Lindeboom and L. M. Bouter | 1999 | Education and incident Alzheimer's disease: a biased association due to selective attrition and use of a two-step diagnostic procedure? | Outcome not HRs |
| Ghassemzadeh, H., A. A. Kamrani, Y. A. Momtaz, M. Rassafiani, F. Nourhashemi, R. Sahaf and S. Naderian | 2019 | OCCUPATIONAL RISK FACTORS FOR DEMENTIA IN A SAMPLE OF OLDER ADULTS COVERED BY THE IRANIAN OIL INDUSTRIES' HEALTH CENTERS, 2018. | Outcome not HRs |
| Griep, Y., L. M. Hanson, T. Vantilborgh, L. Janssens, S. K. Jones and M. Hyde | 2017 | Can volunteering in later life reduce the risk of dementia? A 5-year longitudinal study among volunteering and non-volunteering retired seniors. | Outcome not HRs |
| Guerrero Barragán, A., D. Lucumí and B. Lawlor | 2021 | Association of Leisure Activities With Cognitive Impairment and Dementia in Older Adults in Colombia: A SABE-Based Study. | Outcome not HRs |
| Hack, E. E., J. A. Dubin, M. A. Fernandes, S. M. Costa and S. L. Tyas | 2019 | Multilingualism and Dementia Risk: Longitudinal Analysis of the Nun Study. | Outcome not HRs |
| Håkansson, K., S. Rovio, E.-L. Helkala, A.-R. Vilska, B. Winblad, H. Soininen, A. Nissinen, A. H. Mohammed and M. Kivipelto | 2009 | Association between mid-life marital status and cognitive function in later life: population based cohort study. | Outcome not HRs |
| Hall, K. S., S. J. Gao, F. W. Unverzagt and H. C. Hendrie | 2000 | Low education and childhood rural residence - Risk for Alzheimer's disease in African Americans. | Outcome not HRs |
| Helmer, C., D. Damon, L. Letenneur, C. Fabrigoule, P. Barberger-Gateau, S. Lafont, R. Fuhrer, T. Antonucci, D. Commenges, J. M. Orgogozo and J. F. Dartigues | 1999 | Marital status and risk of Alzheimer's disease - A French population-based cohort study. | Outcome not HRs |
| Helmer, C., L. Letenneur, I. Rouch, S. Richard-Harston, P. Barberger-Gateau, C. Fabrigoule, J. M. Orgogozo and J. F. Dartigues | 2001 | Occupation during life and risk of dementia in French elderly community residents. | Outcome not HRs |
| Hong, X., Z. X. Zhang, Z. Hong, X. H. Liu, J. Qiao, B. Zhou and M. N. Tang | 2004 | Recreational activities, life events and Alzheimer disease. | Outcome not HRs |
| Horikawa, C., R. Otsuka, Y. Nishita, C. Tange, Y. Kato, T. Tanaka, T. Rogi, H. Shibata, F. Ando and H. Shimokata | 2021 | Interaction between cognitive leisure activity and long-chain polyunsaturated fatty acid intake on global cognitive decline in a Japanese longitudinal cohort study: National Institute for Longevity Sciences-Longitudinal Study of Aging. | Outcome not HRs |
| Inzelberg, R., A. E. Afgin, M. Massarwa, E. Schechtman, S. D. Israeli-Korn, R. Strugatsky, A. Abuful, E. Kravitz, L. A. Farrer and R. P. Friedland | 2013 | Prayer at midlife is associated with reduced risk of cognitive decline in Arabic women. | Outcome not HRs |
| Ishtiak-Ahmed, K., Å. M. Hansen, A. H. Garde, E. L. Mortensen, F. Gyntelberg, T. K. T. Phung, R. Lund, N. H. Rod, E. Prescott, G. Waldemar, R. Westendorp and K. Nabe-Nielsen | 2018 | Social Relations at Work and Incident Dementia: 29-Years' Follow-Up of the Copenhagen Male Study. | Outcome not HRs |
| Jia, F., F. Liu, X. Li, X. Shi, Y. Liu and F. Cao | 2021 | Cognitive reserve, modifiable-risk-factor profile and incidence of dementia: results from a longitudinal study of CFAS Wales. | Outcome not HRs |
| Karp, A., R. Andel, M. G. Parker, H.-X. Wang, B. Winblad and L. Fratiglioni | 2009 | Mentally stimulating activities at work during midlife and dementia risk after age 75: follow-up study from the Kungsholmen Project. | Outcome not HRs |
| Karp, A., I. Kåreholt, C. Qiu, T. Bellander, B. Winblad and L. Fratiglioni | 2004 | Relation of education and occupation-based socioeconomic status to incident Alzheimer's disease. | Outcome not HRs |
| Karp, A., S. Paillard-Borg, H.-X. Wang, M. Silverstein, B. Winblad and L. Fratiglioni | 2006 | Mental, physical and social components in leisure activities equally contribute to decrease dementia risk. | Outcome not HRs |
| Kremen, W. S., A. Beck, J. A. Elman, D. E. Gustavson, C. A. Reynolds, X. M. Tu, M. E. Sanderson-Cimino, M. S. Panizzon, E. Vuoksimaa, R. Toomey, C. Fennema-Notestine, D. J. Hagler, Jr., B. Fang, A. M. Dale, M. J. Lyons and C. E. Franz | 2019 | Influence of young adult cognitive ability and additional education on later-life cognition. | Outcome not HRs |
| Laurin, D., R. Verreault, J. Lindsay, K. MacPherson and K. Rockwood | 2001 | Physical activity and risk of cognitive impairment and dementia in elderly persons. | Outcome not HRs |
| Lee, A. T. C., M. Richards, W. C. Chan, H. F. K. Chiu, R. S. Y. Lee and L. C. W. Lam | 2015 | Intensity and Types of Physical Exercise in Relation to Dementia Risk Reduction in Community-Living Older Adults. | Outcome not HRs |
| Lee, A. T. C., M. Richards, W. C. Chan, H. F. K. Chiu, R. S. Y. Lee and L. C. W. Lam | 2018 | Association of Daily Intellectual Activities With Lower Risk of Incident Dementia Among Older Chinese Adults. | Outcome not HRs |
| Letenneur, L., L. J. Launer, K. Andersen, M. E. Dewey, A. Ott, J. R. M. Copeland, J. F. Dartigues, P. Kragh-Sorensen, M. Baldereschi, C. Brayne, A. Lobo, J. M. Martinez-Lage, T. Stijnen and A. Hofman | 2000 | Education and the risk for Alzheimer's disease: Sex makes a difference. EURODEM pooled analyses. | Outcome not HRs |
| Li, C. Y., S. C. Wu and F. C. Sung | 2002 | Lifetime principal occupation and risk of cognitive impairment among the elderly. | Outcome not HRs |
| Li, J., J. J. Llibre-Guerra, A. Harrati, J. Weiss, I. Z. Jiménez-Velázquez, D. Acosta, J. d. J. Llibre-Rodriguez, M.-M. Liu and W. H. Dow | 2021 | Associations between education and dementia in the Caribbean and the United States: An international comparison. | Outcome not HRs |
| Lucca, U., M. Tettamanti, S. Ammesso, M. Garrí, S. Mandelli, E. Riva and A. Recchia | 2016 | Marital status and the risk of dementia in the oldest-old: The monzino 80-plus population-based study. | Outcome not HRs |
| McHugh, J., B. Lawlor, A. Steptoe and F. Kee | 2016 | Interactive impacts of loneliness and social isolation on incident dementia in the english longitudinal study of ageing. | Outcome not HRs |
| Moceri, V. M., W. A. Kukull, I. Emanual, G. van Belle, J. R. Starr, G. D. Schellenberg, W. C. McCormick, J. D. Bowen, L. Teri and E. B. Larson | 2001 | Using census data and birth certificates to reconstruct the early-life socioeconomic environment and the relation to the development of Alzheimer's disease. | Outcome not HRs |
| Moceri, V. M., W. A. Kukull, I. Emanuel, G. van Belle and E. B. Larson | 2000 | Early-life risk factors and the development of Alzheimer's disease. | Outcome not HRs |
| Nabe-Nielsen, K., A. H. Garde, K. Ishtiak-Ahmed, F. Gyntelberg, E. L. Mortensen, T. K. T. Phung, N. H. Rod, G. Waldemar, R. G. J. Westendorp and A. M. Hansen | 2017 | Shift work, long working hours, and later risk of dementia: A long-term follow-up of the Copenhagen Male Study. | Outcome not HRs |
| Nabe-Nielsen, K., Å. M. Hansen, K. Ishtiak-Ahmed, M. B. Grynderup, F. Gyntelberg, S. Islamoska, E. L. Mortensen, T. K. T. Phung, N. H. Rod, G. Waldemar, R. G. J. Westendorp and A. H. Garde | 2019 | Night shift work, long working hours and dementia: a longitudinal study of the Danish Work Environment Cohort Study. | Outcome not HRs |
| Nabe-Nielsen, K., A. Holtermann, F. Gyntelberg, A. H. Garde, S. Islamoska, E. Prescott, P. Schnohr and Å. Hansen | 2021 | The effect of occupational physical activity on dementia: Results from the Copenhagen Male Study. | Outcome not HRs |
| Ngandu, T., E. von Strauss, E. L. Helkala, B. Winblad, A. Nissinen, J. Tuomilehto, H. Soininen and M. Kivipelto | 2007 | Education and dementia: what lies behind the association? | Outcome not HRs |
| O'Donovan, G., M. Hamer, O. L. Sarmiento and P. Hessel | 2020 | Education in early life markedly reduces the probability of cognitive impairment in later life in Colombia. | Outcome not HRs |
| Paillard-Borg, S., L. Fratiglioni, B. Winblad and H. X. Wang | 2009 | Leisure activities in late life in relation to dementia risk: Principal component analysis. | Outcome not HRs |
| Pérès, K., C. Helmer, H. Amieva, J.-M. Orgogozo, I. Rouch, J.-F. Dartigues and P. Barberger-Gateau | 2008 | Natural history of decline in instrumental activities of daily living performance over the 10 years preceding the clinical diagnosis of dementia: a prospective population-based study. | Outcome not HRs |
| Perquin, M., M. Vaillant, A.-M. Schuller, J. Pastore, J.-F. Dartigues, M.-L. Lair and N. Diederich | 2013 | Lifelong exposure to multilingualism: new evidence to support cognitive reserve hypothesis. | Outcome not HRs |
| Podewils, L. J., E. Guallar, L. H. Kuller, L. P. Fried, O. L. Lopez, M. Carlson and C. G. Lyketsos | 2005 | Physical activity, APOE genotype, and dementia risk: Findings from the Cardiovascular Health Cognition Study. | Outcome not HRs |
| Prince, M., D. Acosta, C. Ferri, M. Guerra, Y. Huang, J. L. Rodriguez, A. Salas and A. Sosa | 2011 | The 10/66 dementia research group cohort studies in middle income countries: Dementia incidence and mortality in latin america and China. | Outcome not HRs |
| Qiu, C., L. Bäckman, B. Winblad, H. Agüero-Torres and L. Fratiglioni | 2001 | The influence of education on clinically diagnosed dementia incidence and mortality data from the Kungsholmen Project. | Outcome not HRs |
| Qiu, C., A. Karp, E. von Strauss, B. Winblad, L. Fratiglioni and T. Bellander | 2003 | Lifetime principal occupation and risk of Alzheimer's disease in the Kungsholmen project. | Outcome not HRs |
| Rovio, S., I. Kareholt, E. L. Helkala, M. Viitanen, J. Tuomilehto, A. Nissinen, H. Soininen, B. Winblad and M. Kivipelto | 2005 | Leisure time physical activity at midlife and the risk of dementia and Alzheimer's disease. | Outcome not HRs |
| Rovio, S., I. Kåreholt, M. Viitanen, B. Winblad, J. Tuomilehto, H. Soininen, A. Nissinen and M. Kivipelto | 2007 | Work-related physical activity and the risk of dementia and Alzheimer's disease. | Outcome not HRs |
| Samuel, L. J., S. L. Szanton, J. L. Wolff, K. A. Ornstein, L. J. Parker and L. N. Gitlin | 2020 | Socioeconomic disparities in six-year incident dementia in a nationally representative cohort of US older adults: an examination of financial resources. | Outcome not HRs |
| Sattler, C., P. Toro, P. Schönknecht and J. Schröder | 2012 | Cognitive activity, education and socioeconomic status as preventive factors for mild cognitive impairment and Alzheimer's disease. | Outcome not HRs |
| Sattler, C., P. Toro and J. Schröder | 2009 | Cognitive leisure activity as a protective factor in a prospective ageing study in Germany. | Outcome not HRs |
| Scarmeas, N., G. Levy, M. X. Tang, J. Manly and Y. Stern | 2001 | Influence of leisure activity on the incidence of Alzheimer's disease. | Outcome not HRs |
| Schnaider Beeri, M., R. Ravona-Springer and U. Goldbourt | 2014 | Socioeconomic status and cholesterol levels in midlife and risk of dementia 35 years later. | Outcome not HRs |
| Serra, L., M. Musicco, M. Cercignani, M. Torso, B. Spanò, C. Mastropasqua, G. Giulietti, C. Marra, G. Bruno, G. Koch, C. Caltagirone and M. Bozzali | 2015 | Cognitive reserve and the risk for Alzheimer's disease: a longitudinal study. | Outcome not HRs |
| Sindi, S., G. Hagman, K. Hakansson, J. Kulmala, C. Nilsen, I. Kareholt, H. Soininen, A. Solomon and M. Kivipelto | 2017 | Midlife Work-Related Stress Increases Dementia Risk in Later Life: The CAIDE 30-Year Study. | Outcome not HRs |
| Sindi, S., K. Hakansson, G. Hagman, J. Kulmala, H. Soininen, I. Kareholt, A. Solomon and M. Kivipelto | 2014 | Mid-life work-related stress increases dementia risk in late-life: The caide 30-year study. | Outcome not HRs |
| Skogen, J. C., S. Bergh, R. Stewart, A. K. Knudsen and O. Bjerkeset | 2015 | Midlife mental distress and risk for dementia up to 27 years later: the Nord-Trondelag Health Study (HUNT) in linkage with a dementia registry in Norway. | Outcome not HRs |
| Stern, Y., B. Gurland, T. K. Tatemichi, M. X. Tang, D. Wilder and R. Mayeux | 1994 | Influence of education and occupation on the incidence of Alzheimer's disease. | Outcome not HRs |
| Then, F. S., M. Luppa, M. L. Schroeter, H.-H. Koenig, M. C. Angermeyer and S. G. Riedel-Heller | 2013 | Enriched Environment at Work and the Incidence of Dementia: Results of the Leipzig Longitudinal Study of the Aged (LEILA 75+). | Outcome not HRs |
| Thompson, F., S. G. Russell, L. R. Harriss, A. Esterman, S. Taylor, R. Quigley, E. Strivens and R. McDermott | 2022 | Using Health Check Data to Understand Risks for Dementia and Cognitive Impairment Among Torres Strait Islander and Aboriginal Peoples in Northern Queensland-A Data Linkage Study. | Outcome not HRs |
| Tyas, S. L., M. Iraniparast, M. L. MacKinley, S. M. Costa and D. O. Fearon | 2016 | Academic performance and risk of Alzheimer's disease and dementia in the nun study: Are high grades a protective factor or low grades a risk factor? | Outcome not HRs |
| Arce Rentería, M., Vonk, J.M.J., Felix, G., Avila, J.F., Zahodne, L.B., Dalchand, E., Frazer, K.M., Martinez, M.N., Shouel, H.L., Manly, J.J. | 2019 | Illiteracy, dementia risk, and cognitive trajectories among older adults with low education | Outcome not HRs |
| Tyas, S. L., D. A. Snowdon, M. F. Desrosiers, K. P. Riley and W. R. Markesbery | 2009 | Early-life linguistic ability, late-life pathology and asymptomatic Alzheimer's disease: Findings from the nun study. | Outcome not HRs |
| Valenzuela, M., C. Brayne, P. Sachdev, G. Wilcock and F. Matthews | 2011 | Cognitive lifestyle and long-term risk of dementia and survival after diagnosis in a multicenter population-based cohort. | Outcome not HRs |
| Wang, H.-X., D. R. Gustafson, M. Kivipelto, N. L. Pedersen, I. Skoog, B. Windblad and L. Fratiglioni | 2012 | Education halves the risk of dementia due to apolipoprotein ε4 allele: a collaborative study from the Swedish brain power initiative. | Outcome not HRs |
| Wang, H.-X., A. Karp, B. Winblad and L. Fratiglioni | 2002 | Late-life engagement in social and leisure activities is associated with a decreased risk of dementia: a longitudinal study from the Kungsholmen project. | Outcome not HRs |
| Wang, H.-X., S. W. S. MacDonald, S. Dekhtyar and L. Fratiglioni | 2017 | Association of lifelong exposure to cognitive reserve-enhancing factors with dementia risk: A community-based cohort study. | Outcome not HRs |
| Wang, S., X. Luo, D. Barnes, M. Sano and K. Yaffe | 2014 | Physical activity and risk of cognitive impairment among oldest-old women. | Outcome not HRs |
| Wilson, R. S., C. F. M. de Leon, L. L. Barnes, J. A. Schneider, J. L. Bienias, D. A. Evans and D. A. Bennett | 2002 | Participation in cognitively stimulating activities and risk of incident Alzheimer disease. | Outcome not HRs |
| Wilson, R. S., K. R. Krueger, S. E. Arnold, J. A. Schneider, J. F. Kelly, L. L. Barnes, Y. Tang and D. A. Bennett | 2007 | Loneliness and risk of Alzheimer disease. | Outcome not HRs |
| Wilson, R. S., P. A. Scherr, G. Hoganson, J. L. Bienias, D. A. Evans and D. A. Bennett | 2005 | Early life socioeconomic status and late life risk of Alzheimer's disease. | Outcome not HRs |
| Wilson, R. S., P. A. Scherr, J. A. Schneider, Y. Tang and D. A. Bennett | 2007 | Relation of cognitive activity to risk of developing Alzheimer disease. | Outcome not HRs |
| Wu, Y. T., J. Teale, F. E. Matthews, C. Brayne, B. Woods and L. Clare | 2016 | Lifestyle factors, cognitive reserve, and cognitive function: Results from the Cognitive Function and Ageing Study Wales, a population-based cohort. | Outcome not HRs |
| Yamada, M., H. Sasaki, Y. Mimori, F. Kasagi, S. Sudoh, J. Ikeda, Y. Hosoda, S. Nakamura and K. Kodama | 1999 | Prevalence and risks of dementia in the Japanese population: RERF's Adult Health Study Hiroshima subjects. | Outcome not HRs |
| Yang, S.-Y., P.-H. Weng, J.-H. Chen, J.-M. Chiou, C.-Y. Lew-Ting, T.-F. Chen, Y. Sun, L.-L. Wen, P.-K. Yip, Y.-M. Chu and Y.-C. Chen | 2015 | Leisure activities, apolipoprotein E e4 status, and the risk of dementia. | Outcome not HRs |
| Yeung, C. M., P. D. St John, V. Menec and S. L. Tyas | 2014 | Is bilingualism associated with a lower risk of dementia in community-living older adults? Cross-sectional and prospective analyses. | Outcome not HRs |
| Yusuf, A. J., O. Baiyewu, A. G. Bakari, S. B. Garko, M. E. B. Jibril, A. M. Suleiman, H. M. Muktar and M. A. Amedu | 2018 | Low education and lack of spousal relationship are associated with dementia in older adults with diabetes mellitus in Nigeria. | Outcome not HRs |
| Zahodne, L. B., P. W. Schofield, M. T. Farrell, Y. Stern and J. J. Manly | 2014 | Bilingualism does not alter cognitive decline or dementia risk among Spanish-speaking immigrants. | Outcome not HRs |

# Appendix B. Assessment of risk of bias results

a. Figure B.1. Funnel plots for the association of early-life, middle-life, late-life CR with risk of dementia

B.

A.

C.

The log HRs are plotted against the standard error of the logarithm of the HR. The dashed lines depict the logarithm of the summary HR with its 95% confidence interval. (A) early-life CR. (B) middle-life CR. (C) late-life CR.

HR, hazard ratio; CR, cognitive reserve.

b. Figure B.2. Begg and Egger test for publication bias in studies reporting early-life CR and risk of dementia

c. Figure B.3. Begg and Egger test for publication bias in studies reporting middle-life CR and risk of dementia

d. Figure B.4. Begg and Egger test for publication bias in studies reporting late-life CR and risk of dementia

e. Figure B.5. Trim-and-fill method in studies reporting middle-life and late-life CR and risk of dementia

A.

B.

1. middle-life CR. (B) late-life CR.

# Appendix C. Sensitivity analyses.

Figure C.1. Fixed-effects meta-analysis of risk of dementia according to CR in period of life.

a. Association of early-life CR with risk of dementia in different proxies.

b. Association of middle-life CR with risk of dementia in different proxies.

c. Association of late-life CR with risk of dementia in different proxies.

Figure C.2. Sensitivity analysis given named study is omitted.

a. Association of early-life CR with risk of dementia

b. Association of middle-life CR with risk of dementia

c. Association of late-life CR with risk of dementia
